# A novel c-Kit/phospho-prohibitin axis enhances ovarian cancer stemness and chemoresistance via Notch3—PBX1 and β-catenin—ABCG2 signaling

**DOI:** 10.1186/s12929-020-00638-x

**Published:** 2020-03-13

**Authors:** Chia-Hsun Fang, Yi-Te Lin, Chi-Ming Liang, Shu-Mei Liang

**Affiliations:** 1 Agricultural Biotechnology Research Center, Academia Sinica, 128 Academia Rd, Sec. 2, Taipei, 11529 Taiwan; 2 Institute of Biotechnology, National Taiwan University, 4F, No. 81, Chang-Xing St, Taipei, 10672 Taiwan; 3 Genomics Research Center, Academia Sinica, 128 Academia Rd, Sec. 2, Taipei, 11529 Taiwan

**Keywords:** c-Kit, Prohibitin, Notch3, β-catenin, Cancer stem cells, Lipid raft domain of plasma membrane

## Abstract

**Background:**

The underlying mechanism involved in ovarian cancer stemness and chemoresistance remains largely unknown. Here, we explored whether the regulation of c-Kit and plasma membrane prohibitin (PHB) affects ovarian cancer stemness and chemotherapy resistance.

**Methods:**

Mass spectrum analysis and an in vitro kinase assay were conducted to examine the phosphorylation of PHB at tyrosine 259 by c-Kit. The in vitro effects of c-Kit on membrane raft-PHB in ovarian cancer were determined using tissue microarray (TMA)-based immunofluorescence, western blotting, immunoprecipitation, colony and spheroid formation, cell migration and cell viability assays. In vivo tumor initiation and carboplatin treatment were conducted in nude mice.

**Results:**

We found that c-Kit and PHB colocalized in the raft domain and were positively correlated in human ovarian serous carcinoma. c-Kit interacted with PHB and facilitated the phosphorylation of PHB at tyrosine 259 (phospho-PHB^Y259^) in the membrane raft to enhance ovarian cancer cell motility. The generation of SKOV3GL-G4, a metastatic phenotype of SKOV3 green fluorescent protein and luciferase (GL) ovarian cancer cells, in xenograft murine ascites showed a correlation between metastatic potential and stem cell characteristics, as indicated by the expression of c-Kit, Notch3, Oct4, Nanog and SOX2. Further study revealed that after activation by c-Kit, raft-phospho-PHB^Y259^ interacted with Notch3 to stabilize Notch3 and increase the downstream target PBX1. Downregulation of raft-phospho-PHB^Y259^ increased the protein degradation of Notch3 through a lysosomal pathway and inhibited the β-catenin—ABCG2 signaling pathway. Moreover, raft-phospho-PHB^Y259^ played an important role in ovarian cancer stemness and tumorigenicity as well as resistance to platinum drug treatment in vitro and in vivo.

**Conclusions:**

These findings thus reveal a hitherto unreported interrelationship between c-Kit and PHB as well as the effects of raft-phospho-PHB^Y259^ on ovarian cancer stemness and tumorigenicity mediated by the Notch3 and β-catenin signaling pathways. Targeting the c-Kit/raft-phospho-PHB^Y259^ axis may provide a new therapeutic strategy for treating patients with ovarian cancer.

## Background

Ovarian cancer is the leading cause of death among all gynecological malignancies because of its late diagnosis and high recurrence rate [1]. Although the standard therapy of cytoreductive surgery followed by adjuvant chemotherapy usually results in remission, more than 70% of patients relapse [2]. Exploring the molecular mechanism to develop approaches to prevent ovarian tumor recurrence is an unmet clinical need.

One promising approach is to study the survival signaling pathways of tumor initiating cancer stem cells (CSCs) that possess the ability to self-renew, differentiate and regenerate tumors in vivo. These CSCs are generally resistant to conventional cancer therapies and are positively regulated in tumor metastasis and recurrence [3–5]. Hence, targeting CSCs could improve current cancer treatment and help decrease the rate of ovarian cancer relapse. The most commonly used markers for the isolation of ovarian cancer CSCs are CD44, CD133, CD24, CD117 (c-Kit) and ALDH1A1, which are often used in combination [6–11]. In addition, pluripotent stem cell markers of normal stem cells, notably OCT4, NANOG and SOX2, have also been found to play an important role in ovarian cancer development and metastasis and can be used to verify cell stemness [12].

Seventy percent of epithelial ovarian cancers are high-grade serous adenocarcinomas, which often contain ascites [13]. The accumulation of malignant ascites in the peritoneal cavity provides a microenvironment favorable for the formation of spheroids that are enriched in stem cells [14, 15]. Recently, abnormal c-Kit overexpression but not c-Kit mutations have been observed in ovarian serous carcinoma and correlated with a shorter survival time [16–18]. It has been found that c-Kit can mediate drug resistance and play an important role in regulating ovarian tumor-initiating cell survival and proliferation through the Wnt/β-catenin—ABCG2 pathway [19]. Stem cell factor (SCF) binds to c-Kit to activate PI3K/Akt, SRC, PLCγ1, JAK/STAT, RAS/MAP kinase, Wnt/β-catenin and Notch pathways, which drive the proliferation, survival, migration and stemness phenotype of cancer [8, 19–22]. However, whether c-Kit needs other cofactor(s) or mediator(s) to activate these pathways is unclear.

Prohibitin (PHB, also known as PHB1) is a highly conserved and ubiquitously expressed protein localized to different cellular compartments, including the lipid raft of the plasma membrane, mitochondria, cytoplasm or nucleus, and has diverse biological functions [23, 24]. In A549 lung cancer cells, an increase in surface PHB expression has been shown to prevent the apoptosis of cancer cells and render them more resistant to paclitaxel [25]. It has also been found that overexpression of PHB results in resistance to diverse chemotherapeutic drugs in Wilms’ tumor [26]. In neuroblastoma, PHB regulates cancer cell self-renewal and clonogenic potential [27, 28]. Our lab previously demonstrated that phosphorylation of PHB at Y259 (phospho-PHB^Y259^) and T258 (phospho-PHB^T258^) in the lipid raft of the plasma membrane activates Raf-1, which subsequently activates ERK and promotes cancer development [29]. Phospho-PHB^Y259^ regulates phospho-PHB^T258^ but not vice versa [29]. However, it is unclear which tyrosine kinase facilitates the formation of phospho-PHB^Y259^ in the lipid raft domain of the plasma membrane to initiate the biological functions of phospho-PHB^Y259^.

In this study, we showed that c-Kit associates with PHB to upregulate phospho-PHB^Y259^ in the lipid raft of the plasma membrane. There is a positive correlation between c-Kit and phospho-PHB^Y259^ in advanced serous ovarian carcinoma. Phosphorylation of PHB by c-Kit promotes ovarian cancer stem cell survival and proliferation through activation of not only the β-catenin signaling pathway but also the Notch3 signaling pathway.

## Materials and methods

### Materials

Imatinib mesylate (sc-202,180, Santa Cruz Biotechnology, Dallas, TX, USA) was dissolved in double-distilled H_2_O to produce a 100 mM stock solution and used to treat cells at different concentrations for 24 h. Carboplatin (S1215, Selleckchem, Houston, TX, USA) was dissolved in double-distilled H_2_O to produce a 10 mM stock solution and then used to treat cells at different concentrations for 72 h. Cycloheximide (C4859, MilliporeSigma, Burlington, MA, USA) was dissolved in DMSO to produce a 100 mg/ml stock solution and used to treat cells at 50 μM at the indicated time points. Chloroquine diphosphate (S4157, Selleckchem) was dissolved in double-distilled H_2_O to produce a 50 mM stock solution and used to treat cells at 25 μΜ for 24 h. MG132 (M7449, MilliporeSigma) was dissolved in DMSO to produce a 10 mM stock solution and used to treat cells at 20 μΜ for 7 h. Primary antibodies against the following antigens were used in western blot analysis: c-Kit (3074, Cell Signaling Technology, Danvers, MA, USA), PHB (GTX101105, GeneTex, Irvine, CA, USA), phospho-PHB^Y259^ (11,587, SAB; Signalway Antibody, College Park, MD, USA), phospho-PHB^T258^ (11,588, SAB; Signalway Antibody), caveolin 1 (GTX100205, GeneTex), clathrin heavy chain (2410, Cell Signaling Technology), E-cadherin (20874–1-AP, Proteintech, Chicago, IL, USA), N-cadherin (GTX127345, GeneTex), ZEB1 (GTX105278, GeneTex), Notch3 (5276, Cell Signaling Technology), PBX1 (GTX113242, GeneTex), β-catenin (GTX101435, GeneTex), ABCG2 (GTX100437, GeneTex) and β-actin (E1C605, EnoGene, Nanjing, China).

### Cells and cell culture

The human ovarian cancer cell lines SKOV3 and KURAMOCHI were purchased from American Type Culture Collection (Manassas, VA, USA) and the Japanese Collection of Research Bioresources Cell Bank (Osaka, Japan), respectively. KURAMOCHI cells were maintained in RPMI-1640 medium (22,400,089, Gibco, Thermo Fisher Scientific, Waltham, MA, USA) supplemented with 10% fetal bovine serum (HyClone, Logan, UT, USA), 100 units/ml penicillin and 100 μg/ml streptomycin in a 37 °C incubator with a humidified atmosphere containing 5% CO_2_. SKOV3GL cells that express both green fluorescent protein (G) and luciferase (L) were previously generated in our lab [30]. To generate more metastatic phenotype(s) of SKOV3, SKOV3GL cells were injected into nude mouse ovaries until cells metastasized to the abdominal cavity (ascites), and cells were isolated from ascites to obtain SKOV3GL-G1 cells. The same procedure was repeated several times to obtain SKOV3GL-G2 from SKOV3GL-G1, SKOV3GL-G3 from SKOV3GL-G2, and SKOV3GL-G4 from SKOV3GL-G3. All of these phenotypes of SKOV3 cells were maintained in McCoy’s 5A medium (16,600,082, Gibco) supplemented with 10% fetal bovine serum (HyClone), 100 units/ml penicillin and 100 μg/ml streptomycin.

### Plasmid constructs, reagents and transfection

Plasmid *pD-PHB-HA* was generated by fusing the PHB gene at the C-terminus to the PDGFR transmembrane domain and tagged with the HA epitope at the N-terminus as described in our previous publication [29]. The PHB mutant *pD-PHB*^*Y259F*^*-HA* was produced using the QuikChange Site-directed Mutagenesis Kit (Stratagene, La Jolla, CA, USA) according to the manufacturer’s instructions. Cells were transiently transfected with *pD-PHB-HA* or *pD-PHB*^*Y259F*^*-HA* plasmids for 48 h using TransIT-X2 (Mirus Bio, Madison, WI, USA).

Plasmid *pLVX-IRES-****c-Kit****-Puro* with a tagging c-Myc epitope at the C-terminus was purchased from Biotools (New Taipei, Taiwan). The lentivirus system was used to transfect *pLVX-IRES-****c-Kit****-Puro* plasmid into SKOV3 cells to establish an SKOV3_c-Kit stable clone according to the protocol of the National RNAi Core Facility, Academia Sinica, Taipei, Taiwan.

### c-Kit siRNA transfection

SKOV3GL-G4 or KURAMOCHI cells were cultured to 80% confluence and transiently transfected with a negative scramble control siRNA (sc-37,007, Santa Cruz Biotechnology) or anti-c-Kit siRNA (sc-29,225, Santa Cruz Biotechnology), including a pool of four designed target-specific 19–25 nt siRNAs, to knockdown c-Kit gene expression by using TransIT-X2.

### In vitro kinase assays and mass spectrum analysis

C-Kit kinase activity was assessed using the c-Kit Kinase Enzyme System (V4498, Promega, Madison, WI, USA) + ADP-Glo Kinase Assay Kit (V9101, Promega) according to the manufacturer’s instructions to measure ADP production in kinase reactions. Recombinant PHB protein (1 μg) (137,155, USBiological, Salem, MA, USA) was incubated with 0–160 ng of recombinant kinase domain of c-Kit. The kinase reaction with a final ATP concentration of 50 μM was incubated at room temperature for 3 h. The reaction mixture was then terminated by adding ADP-Glo reagent for 40 min, followed by the addition of kinase detection reagent for 30 min incubation before reading the luminescence on a multimode microplate reader (Biotek Synergy H1 with 2Di, Winooski, VT, USA). For in vitro kinase detection by immunoblotting, 1 μg of recombinant PHB protein and 0–160 ng recombinant kinase domain of c-Kit were incubated together using the abovementioned buffers. Proteins in the kinase reaction were denatured and reduced using SDS-PAGE sample buffer supplemented with 10% (v/v) β-mercaptoethanol and subjected to immunoblotting to detect phosphorylation of PHB and c-Kit. A pre-absorption experiment was performed as a negative control. Phospho-PHB^Y259^ antibody was incubated with an excess amount of the phosphopeptide spanning phospho-tyrosine 259 of humane PHB (10 μg peptide/1 μg antibody) for 2 h at room temperature, prior to adding to the blots. The sequence of the phosphopeptide is IAYQLSRSRNIT (pY) LPAGQSVLLQ (EnoGene).

For mass spectrum analysis, dithiothreitol (DTT, final 20 mM) was added to the reaction mixture from the ADP-Glo Kinase Assay and incubated at 60 °C for 1 h, and then iodoacetamide (final concentration 50 mM) was added for 45 min in the dark. Then, the pH value was adjusted to 7.0, and samples were digested with trypsin overnight at 37 °C. These digested peptides were analyzed using LC/MS/MS in the Proteomics Core Facility, Institute of Biomedical Sciences, Academia Sinica, Taipei, Taiwan.

### Western blot analysis

Equivalent amounts of proteins from each sample were separated by SDS-PAGE and transferred onto a polyvinylidene difluoride membrane (GE Healthcare, Waukesha, WI, USA). Membranes were blocked in 5% skim milk for 30 min and incubated with primary antibody at 4 °C overnight. After being washed with 1× Tris-buffered saline with Tween 20 (TBST), membranes were incubated with horseradish peroxidase (HRP)-conjugated secondary antibody, rabbit IgG-HRP (GTX213110–01, GeneTex) or mouse IgG-HRP (5450–0011, SeraCare KPL, Milford, MA, USA), at room temperature for 90 min. Membranes were washed three times in 1× TBST for 10 min. Peroxidase activity was detected using chemiluminescence by Luminata Forte Western HRP substrate (Millipore, Billerica, MA, USA). β-actin was used as an internal loading control.

### Isolation of membrane raft protein

Cells were washed in ice-cold PBS and lysed by incubation for 30 min in ice-cold lysis buffer (0.5% Triton X-100, 150 mM NaCl, 20 mM Tris-HCl, pH 7.5) containing proteinase and phosphatase inhibitor cocktail (Roche, Basel, Switzerland). After centrifugation at 16,000 g for 30 min at 4 °C, the supernatants were collected and referred to as the cytosolic plus non-raft membrane (C + M) fraction. The insoluble pellets were resuspended in the same lysis buffer supplemented with 0.5% SDS and 2 mM DTT and sonicated for 10 min at 4 °C. After centrifugation at 16,000 g for 30 min at 4 °C, the supernatants consisting of membrane raft proteins were collected and analyzed by western blotting.

### Immunoprecipitation

Cells were washed twice with Dulbecco’s phosphate-buffered saline (DPBS) and lysed with RIPA buffer (Millipore) containing proteinase and phosphatase inhibitor cocktail (Roche) on ice for 30 min. The cell lysates (500–1000 μg) were incubated with 5 μg anti-HA antibody (51064–2-AP, Proteintech) and 50 μl protein G mag beads (GE Healthcare) on a rotating device at 4 °C overnight. Subsequently, pellets were washed three times with DPBS. Immunoprecipitated proteins were separated by 10% SDS-PAGE and transferred onto a polyvinylidene difluoride membrane (GE Healthcare). The membrane was blocked in 5% skim milk for 30 min and incubated with primary antibodies at 4 °C overnight. After being washed with TBST buffer, the membrane was incubated with HRP-conjugated secondary antibody at room temperature for 2 h. Peroxidase activity was detected using chemiluminescence by Luminata Forte Western HRP substrate (Millipore).

### Reverse transcription and real-time PCR

Total RNA was extracted with the RNeasy Mini Kit (QIAGEN, Hilden, Germany), and 2 μg RNA was reverse transcribed with the ToolsQuant II Fast RT Kit (Biotools). The sequences of the specific primers are provided in Supplementary Information, Table S2. Real-time polymerase chain reaction analysis was set up with SYBR Green qPCR Master Mix (Thermo) and carried out in an ABI Prism 7500 Fast Detection System (Applied Biosystems, Foster City, CA, USA). The relative level of mRNA expression of each gene was determined by normalizing with GAPDH, and the fold increase in the signal over that derived from parental samples was determined with the ΔΔCт calculation.

### Transwell migration assay

Optimal numbers of cells suspended in 100 μl of serum-free medium were seeded into Transwell polycarbonate membrane inserts with 8-μm pores (Corning, Corning, NY, USA) for the migration assay. Complete growth medium containing 10% FBS was added into the lower chamber as the chemoattractant. After incubation for 24 h, cells that migrated through the membrane from the upper to the lower side were fixed by immersing the filter in methanol for 10 min; then, the cells were stained with Liu’s stain A and B reagent (TonYar Biotech, Taoyuan, Taiwan) and counted under a microscope at 200X magnification. Each experiment contained triplicates for each condition, and three independent experiments were carried out for each group.

### Cell viability assay

Cytotoxicity was estimated using the WST assay. Cells were seeded in 6-cm plates at a density of 8 × 10^5^ cells/plate. The next day, cells were transfected with *pDisplay, pD-PHB-HA* or *pD-PHB*^*Y259F*^*-HA* plasmid and incubated for 48 h. Transfected cells were then replated in a 96-well culture plate at a density of 5 × 10^3^ cells/well and treated with serial dilutions of carboplatin. Cell viability was measured after 72 h with CCK-8 solution (Dojindo, Kumamoto, Japan) using a microplate reader (BioTek ELx800). The IC_50_ was defined as the concentration of carboplatin that resulted in a 50% decrease in the number of live cells.

### Colony forming assay

Tumor cells were plated at a density of 300 cells/well in 6-well plates. After 14 days, colonies were fixed with 4% paraformaldehyde for 10 min and visualized with crystal violet (HT-90132, Sigma-Aldrich, St Louis, MO, USA). Clonogenic efficiency = number of colonies (≥50 cells/colony) per input cell × 100%.

### Spheroid formation assay

In total, 200 single viable cells were plated on ultralow attachment six-well plates (Corning) and cultured in serum-free DMEM/F12 medium (11,330,032, Gibco) supplemented with N_2_ (17,502,048, Gibco), 20 ng/mL human recombinant epidermal growth factor (BMS320, eBioscience, San Diego, CA, USA), and 20 ng/mL basic fibroblast growth factor (68–8785-39, eBioscience) for approximately 2 weeks. Representative photos were taken on days 4, 8, and 12. Spheroid sizes were digitally determined and normalized to the size measured on day 4.

### Fluorescence histochemistry for detection of PHB and c-Kit in the plasma membrane of human ovarian cancer tissues

Human ovarian cancer tissue microarray (TMA) slides OV807 and OV803b were purchased from US Biomax (Rockville, MD, USA). TMA samples contained normal ovarian tissues and various types of ovarian carcinoma, including pathological grade, clinical stage, and tumor-node-metastasis classification. Here, we selected normal ovarian tissues from OV807 and serous-type ovarian carcinoma from OV803b for analysis. In brief, all tissue samples were fixed with formalin and embedded in paraffin. After deparaffinizing with xylene, rehydrating with alcohol and retrieving antigen, the slides were blocked with 1% BSA for 1 h at room temperature and incubated with anti-c-Kit antibody (1:200) (3074, Cell Signaling Technology) and 1 μM rhodamine-tagged CKGGRAKDC peptide (Genemed Synthesis, San Antonio, TX, USA), which stains for PHB in the dark at room temperature for 2 h. After being washed with PBS, the slides were incubated with secondary antibody (anti-Rb Alexa 488, A11008, Invitrogen, Eugene, OR, USA) at room temperature for 1 h to detect c-Kit. After being washed with PBS, the slides were mounted with DAPI Fluoromount-G (Southern Biotech, Birmingham, AL, USA). The slides were viewed under a confocal microscope (Zeiss LSM 780 + ELYRA, Carl Zeiss, Jena, Germany), and the cellular distribution of PHB and c-Kit throughout the cell was analyzed using ZEN software. The ratio of plasma membrane to cytoplasmic fluorescence intensity (Ipm/Icyt) in ten cells of each tissue sample was used to quantitate the extent of membrane colocalization of PHB and c-Kit.

### Immunofluorescence

SKOV3_c-Kit cells were seeded on cover slips in 12-well plates. Cells were fixed with 4% paraformaldehyde and incubated with primary antibodies against Notch3 mouse monoclonal antibody (NBP2–52521, Novus Biologicals, Littleton, CO, USA) or c-Kit rabbit polyclonal antibody (3074, Cell Signaling Technology) and coincubated with 1 μM rhodamine-tagged CKGGRAKDC peptide (Genemed Synthesis, San Antonio, TX, USA) that stained for raft-PHB at 4 °C overnight. After being washed with PBS, the cells were stained with Alexa Fluor 488 (anti-Ms or anti-Rb) or DyLight 650 (anti-Rb, A120-201D5, Bethyl Laboratories, Montgomery, TX, USA)-conjugated secondary IgG antibodies at room temperature for 1 h. DAPI Fluoromount-G (Southern Biotech) was used to mount the slides after staining for specific proteins was completed. Notch3 (green), raft-PHB (red), c-Kit (green or pink) and nuclei (blue) were detected with a Zeiss LSM 780 confocal microscope (Carl Zeiss).

### In vivo tumor initiation and chemoresistance assay

Female athymic nude mice (6–8 weeks old) were purchased from the National Laboratory Animal Center, Taiwan, and the animal experiments were approved by the Institutional Animal Care and Utilization Committee of Academia Sinica, Taiwan. For the tumorigenicity assay on the flanks of mice, various numbers (10^5^, 10^3^, and 500) of sphere cells were suspended in DPBS medium, mixed with Matrigel (354,234, BD Biosciences, San Jose, CA, USA) at a 1:1 ratio and then transplanted subcutaneously into nude mice. Tumor formation was monitored by an IVIS imaging system and quantified using Living ImageR 2.50 software. In the chemoresistance assay, SKOV3GL-G4 cells (2 × 10^6^) were transfected with empty vector or *pD-PHB*^*Y259F*^*-HA* and subcutaneously injected into athymic nude mice. When palpable tumors developed, mice were randomized into four groups (*n* = 5 for each group): two groups were injected with SKOV3GL-G4 cells transfected with empty vector and the other two were injected with SKOV3GL-G4 cells transfected with *pD-PHB*^*Y259F*^*-HA*. In the experiment of treatment with carboplatin, 10 mg/ml carboplatin was dissolved in ddH_2_O and administered by intraperitoneal injection (30 mg/kg) three times per week. Tumor volume was measured using digital calipers and calculated with the formula: volume = 1/2(length×width^2^). Tumor weight was recorded after animals were sacrificed. After mice were killed, xenograft tumors were excised and fixed in formalin for further analysis.

### Kaplan-Meier curves

The Kaplan-Meier online plotter tool (http://kmplot.com/analysis/) was used to generate overall survival curves of *c-Kit* and *PHB* from serous ovarian cancer patients according to the autoselected best cutoff values by the plotter tool.

### Statistical analysis

Statistical comparisons between two groups were made using two-tailed Student’s *t* test and among groups of more than three with analysis of variance (ANOVA) followed by post hoc multiple comparisons (Duncan multiple-range test) among means. Correlation data were determined by using Pearson correlation coefficients. Differences between groups were considered statistically significant at *P* < 0.05 (*), *P* < 0.01 (**), *P* < 0.001 (***) and *P* < 0.0001 (****).

## Results

### c-Kit phosphorylates PHB at the Tyr259 residue

**Fig. 1:**
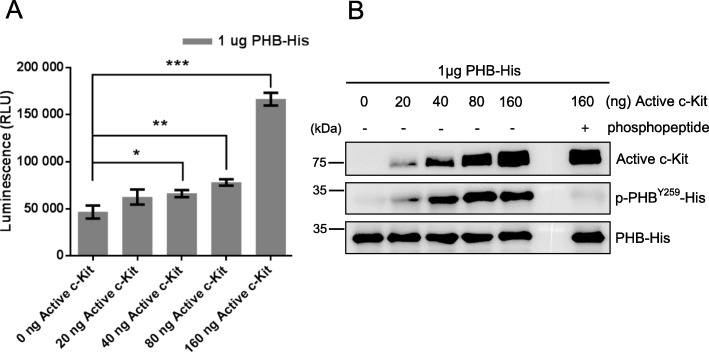
c-Kit phosphorylates PHB at tyrosine 259. **a** Different amounts of active c-Kit kinase domain (0, 20, 40, 80, 160 ng) reacted with 1 μg PHB-His in an in vitro kinase assay. The luminescence was read using a Biotek microplate reader. Data represent the mean ± SD of three independent experiments. ***, *P* < 0.001; **, *P* < 0.01; *, *P* < 0.05, *t* test. **b** Activity of c-Kit kinase, phospho-PHB^Y259^-His and PHB-His from the in vitro kinase assay was analyzed by western blotting. For preabsorption control, anti-phospho-PHB^Y259^ antibody was pre-incubated with an excess amount of the phosphopeptide spanning phospho-tyrosine 259 of PHB (IAYQLSRSRNIT (pY)LPAGQSVLLQ; 10 μg peptide/1 μg antibody) for 2 h at room temperature and then used for western blotting in the rightmost lane. Blots are representative of three independent experiments

To identify which tyrosine kinase increases the phosphorylation of PHB to form phospho-PHB^Y259^, we used the online software Group-based Prediction System (GPS) for prediction. Based on the score and cut-off, c-Kit was identified as the most likely candidate protein (Suppl. Fig. 1). To verify the prediction, we performed an ADP-Glo kinase assay and showed that c-Kit (> 40 ng) phosphorylated PHB in a dose-dependent manner, as indicated by the increase in luminescence (Fig. 1a). We next analyzed phospho-PHB by liquid chromatography-tandem mass spectrometry (LC/MS/MS). According to the MS/MS spectrum, we found that tyrosine 259 of PHB was indeed phosphorylated by c-Kit (Suppl. Fig. 2). This result was further substantiated by immunoblotting detection of phospho-PHB^Y259^ in an in vitro kinase assay, as shown in Fig. 1b. Preabsorption of the anti-phospho-PHB^Y259^ antibody with phosphopeptide spanning phospho-tyrosine 259 of PHB nullified the immunolabeling of phospho-PHB^Y259^ (Fig. 1b). These results indicate that c-Kit directly phosphorylates PHB at tyrosine 259 in vitro.

### c-Kit and PHB are positively correlated and colocalized in the membrane raft domain of ovarian serous carcinoma

**Fig. 2:**
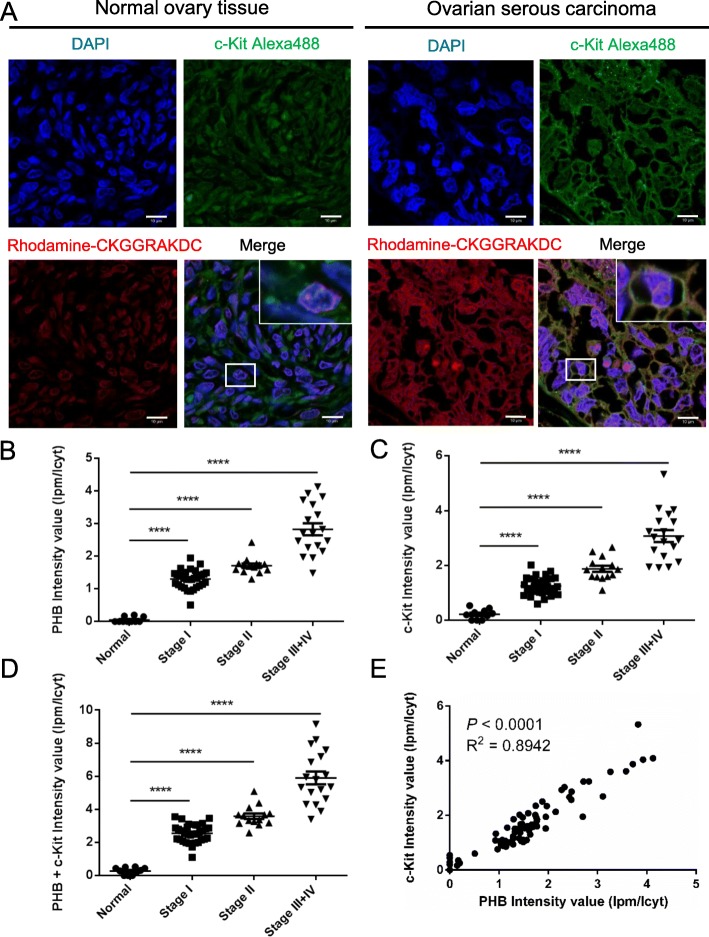
Colocalization of c-Kit and PHB is positively correlated in the membrane raft domain of ovarian serous carcinoma. **a** Human ovarian tissue sections were stained using rhodamine-tagged CKGGRAKDC (red) for PHB, Alexa Fluor 488 (green) for c-Kit and DAPI (blue) for nuclei to detect colocalization of PHB and c-Kit by confocal fluorescence microscopy. The rhodamine-tagged CKGGRAKDC peptide is known to bind PHB in adipose endothelial cells. Scale bar = 10 μm. **b, c** and **d** The graph indicates the levels of plasma membrane-associated PHB and c-Kit at different stages of ovarian serous carcinoma (Normal: *n* = 10; Stage I: *n* = 32; Stage II: *n* = 14; Stage III + IV: *n* = 18). Ipm: fluorescence intensity at the plasma membrane; Icyt: fluorescence intensity at the cytoplasm. Data were analyzed using one-way ANOVA followed by Dunnett’s test; horizontal bars indicate the means ± SEM. **e** Scatter plot shows the correlation of c-Kit with PHB. R: Pearson correlation coefficients; R^2^ means ‘the goodness of fit’. Statistical significance was calculated by Pearson correlation coefficients. ****, *P* < 0.0001

Kaplan-Meier curves showed that high levels of *c-Kit* and *PHB* correlated with shorter overall survival rates in 150 ovarian serous carcinoma patients using the KM-Plotter database (Suppl. Fig. 3). Furthermore, both plasma membrane-associated PHB and abnormal c-Kit expression have been demonstrated to be associated with cancer invasiveness [16, 17, 21, 29]. To examine the relationship between c-Kit and plasma membrane-associated PHB, we analyzed human ovarian serous carcinoma tissues (Suppl. Table 1) with confocal immunofluorescence for the distribution of plasma membrane-associated PHB and c-Kit at different clinical stages. For this analysis, we used the rhodamine-tagged peptide CKGGRAKDC, which is known to selectively bind PHB in adipose endothelial cells and the cell surface of drug-resistant lung cancer cells, [25, 31, 32] to stain for PHB in the membrane raft domain and Alexa 488 to stain for c-Kit. Our results showed that c-Kit and PHB colocalized in the membrane raft domain of ovarian serous carcinoma but not in normal ovarian tissue (Fig. 2a). We further measured the ratio of fluorescence intensity of the plasma membrane (Ipm) to cytoplasm (Icyt) to quantitate the extent of membrane colocalization of c-Kit and PHB (Suppl. Fig. 4). The quantification results showed that the ratio of intensity (Ipm/Icyt) of PHB or c-Kit was higher in ovarian serous carcinoma than in normal ovary tissue and gradually increased from clinical stage I to stage III + IV (*P* < 0.0001) (Fig. 2 b and c). The ratio of PHB + c-Kit intensity was also sequentially enhanced from clinical stage I to stage III (Fig. 2d). Importantly, c-Kit overexpression was accompanied by higher levels of PHB in the membrane raft domain, whereas lower c-Kit expression was observed with lower raft-PHB, indicating a positive correlation between PHB and c-Kit (*P* < 0.0001; Fig. 2e). c-Kit and PHB were thus colocalized in the membrane raft domain and positively correlated in human ovarian serous carcinoma.

### c-Kit phosphorylates PHB in the membrane raft to enhance cancer cell motility

**Fig. 3:**
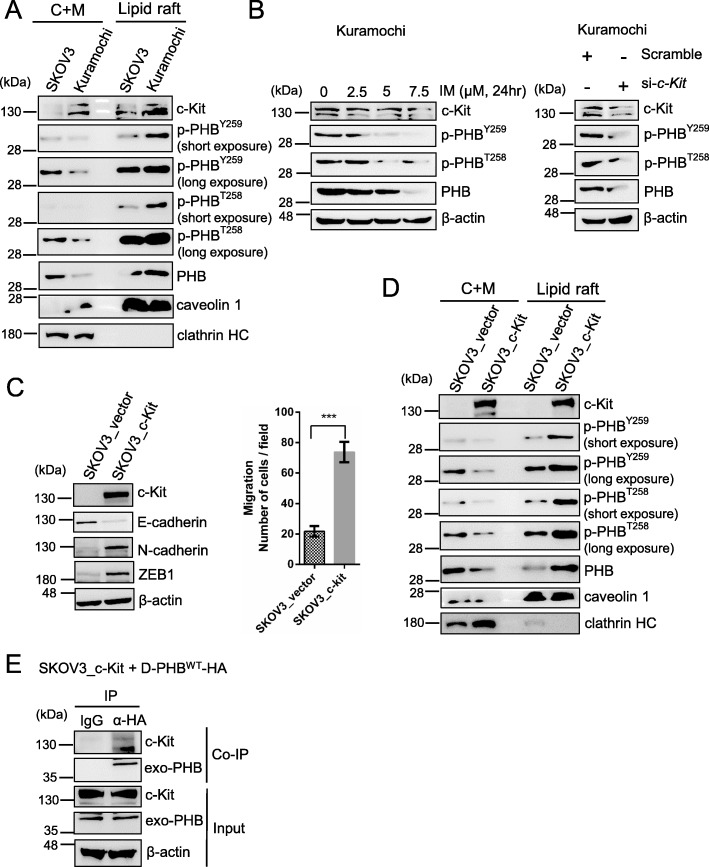
c-Kit phosphorylates PHB in the membrane raft domain to enhance cancer cell migration. **a** Western blot analysis of c-Kit and PHB expression in the membrane raft or cytosolic plus non-raft membrane (C + M) fraction of SKOV3 and KURAMOCHI ovarian cancer cells. Caveolin-1 and clathrin heavy chain (HC) proteins are membrane raft and non-raft markers, respectively. **b** KURAMOCHI cells were treated with c-Kit inhibitor, imatinib mesylate (IM) for 24 h or siRNA for 72 h. Total cell lysates were subjected to western blotting. **c** SKOV3 cells transduced with lentiviral empty vector or vector encoding c-Kit were analyzed by western blotting of EMT markers. The migration ability was determined by 24-h Boyden chamber assay. Data represent the mean ± SD of three independent experiments. ***, *P* < 0.001, *t* test. **d** Analysis of c-Kit and PHB expression in the membrane raft or cytosolic plus non-raft membrane (C + M) fraction of SKOV3 and SKOV3_c-Kit cancer cells. **e** A SKOV3_c-Kit stable clone was transiently transfected with plasmid *pD-PHB*^*WT*^*-HA* that expresses exogenous PHB only in the membrane raft. The cell extracts were immunoprecipitated with anti-HA tag antibody, and coimmunoprecipitated proteins were detected by western blotting with anti-PHB and anti-c-Kit antibodies. Blots are representative of three independent experiments

To further elucidate the relationship between c-Kit and PHB, we analyzed the levels of c-Kit, phospho-PHB and PHB in the lipid raft and non-lipid raft (cytosolic plus non-raft membrane) domains of SKOV3 and KURAMOCHI ovarian cancer cells. KURAMOCHI cells were more clinically related to human high-grade serous ovarian carcinoma than SKOV3 cells [33]. The expression levels of c-Kit, phospho-PHB^Y259^ and phospho-PHB^T258^ in the KURAMOCHI lipid raft domain were higher than those in the SKOV3 lipid raft domain (Fig. 3a right). Conversely, in the non-raft domain (C + M), the levels of phospho-PHB and PHB were lower in KURAMOCHI cells than in SKOV3 cells, whereas c-Kit expression was also higher in KURAMOCHI cells (Fig. 3a left). Inhibition of c-Kit upregulation in KURAMOCHI cells by treatment with imatinib mesylate (2.5–7.5 μM), a clinically used c-Kit inhibitor, or c-Kit siRNA (80 nM) decreased phospho-PHB^Y259^, phospho-PHB^T258^ and PHB expression (Fig. 3b). Furthermore, we established an SKOV3-overexpressing c-Kit stable clone (SKOV3_c-Kit) using the lentivirus system. We found that overexpression of c-Kit in SKOV3 cells not only enhanced epithelial mesenchymal transition (EMT) and migration ability (Fig. 3c) but also increased phospho-PHB^Y259^, phospho-PHB^T258^, and PHB expression in the lipid raft domain (Fig. 3d). Overexpression of c-Kit, on the other hand, reduced PHB expression in the non-raft domain (C + M) to a level similar to that in KURAMOCHI cells (Fig. 3a left). To further assess whether c-Kit interacts with PHB in the lipid raft domain, SKOV3_c-Kit cells were transiently transfected with *pD-PHB*^*WT*^*-HA* to express exogenous PHB in the membrane raft. Immunoprecipitation assays with anti-hemagglutinin (HA)-tagged antibodies showed that c-Kit interacted with PHB in the lipid raft domain of SKOV3_c-Kit cells (Fig. 3e). These results indicate that c-Kit interacts with PHB to phosphorylate PHB in the membrane raft domain, leading to an increase in cancer cell migration.

### Metastatic phenotypes of SKOV3 in xenograft murine ascites show a positive correlation between membrane raft-associated PHB and stem cell characteristics

**Fig. 4:**
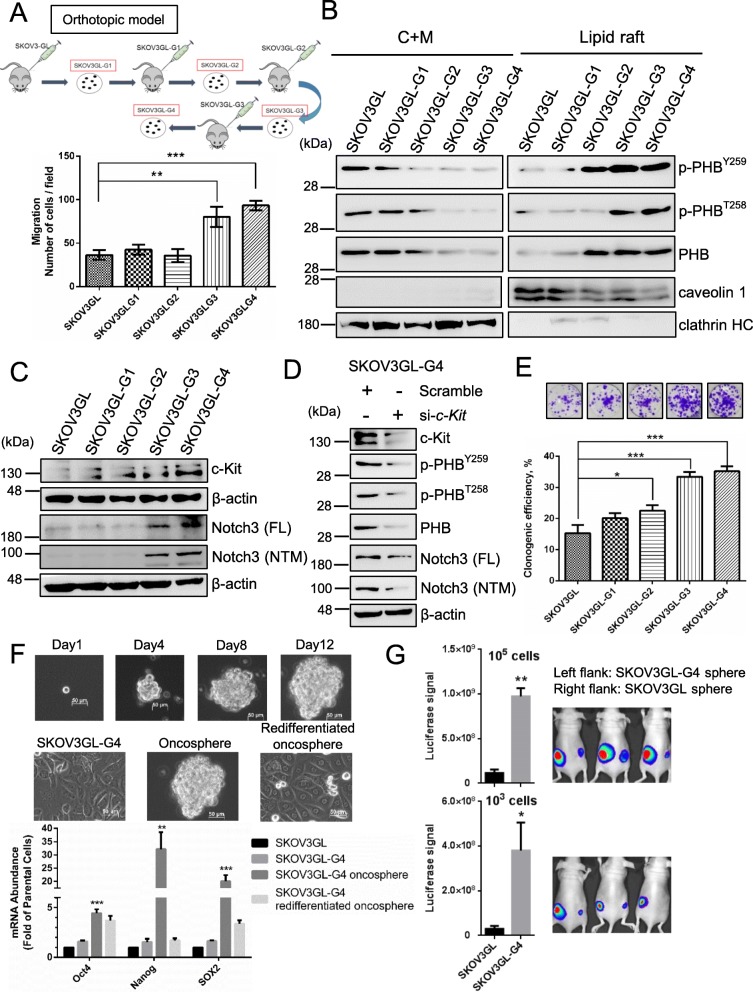
Metastatic ovarian cancer cells enhance c-Kit, Notch3, and membrane raft-associated PHB and contribute to CSC enrichment. **a** Production of metastasized ovarian cancer cells from several passages of SKOV3GL cells into the mouse intraperitoneal cavity. The migration ability increased in SKOV3GL-G3 and SKOV3GL-G4 cells as determined by a 24-h Boyden chamber assay. **b** The levels of phospho-PHB^Y259^, phospho-PHB^T258^, and PHB in the lipid raft or cytosolic plus non-raft membrane (C + M) fraction of SKOV3GL, SKOV3GL-G1 to -G4 cells were detected by western blotting. **c** Expression of c-Kit, full-length (FL) Notch3 and membrane tethered Notch3 fragment (NTM) were analyzed from SKOV3GL to SKOV3GL-G4 cells by western blotting. **d** SKOV3GL-G4 cells were transfected with scrambled control or c-Kit siRNA for 48 h. Total cellular c-Kit, Notch3 (FL & NTM), phospho-PHB, PHB and β-actin protein expression was analyzed by western blotting. **e** Colony forming assay. SKOV3GL and SKOV3GL-G1 to -G4 cells were plated at a density of 300 cells/well in 6-well plates. **f** Phase contrast photomicrographs showing clonal expansion of SKOV3GL-G4 cells into the oncosphere over a 12-day period (top). Photomicrographs of SKOV3GL-G4 adherent cells, oncosphere expansion in low-adherence culture, and redifferentiated oncosphere cells after return to adherent culture (middle). qPCR for putative stem cell markers (Oct4, Nanog, SOX2) expressed as fold of SKOV3GL parental cells (bottom). Scale bar = 50 μm. All statistics represent the mean ± SD of three independent experiments. ***, *P* < 0.001; **, *P* < 0.01; *, *P* < 0.05, *t* test. Blots are representative of three independent experiments. **g** SKOV3GL and SKOV3GL-G4 cells were seeded in CSC medium for 12 days. After 12 days, spheres were harvested and trypsinized into single cells. A total of 1000 or 100,000 cells in 50 μl DPBS were mixed with 50 μl Matrigel and subcutaneously injected into the flanks of nude mice. In vivo CSC properties were determined by luciferase signal at week 4 post injection (mean ± SD; n = 3 per group; **, *P* < 0.01; *, *P* < 0.05, *t* test)

Since ovarian cancer often metastasizes to the intraperitoneal cavity to produce ascitic fluids in patients, we created a murine ascitic model. SKOV3 cells expressing green fluorescent protein and luciferase (SKOV3GL) were used as parental cells in this murine ascitic model to generate several generations of metastatic phenotypes of SKOV3, namely, SKOV3GL-G1, SKOV3GL-G2, SKOV3GL-G3 and SKOV3GL-G4 (Fig. 4a, top). Migration ability significantly increased in SKOV3GL-G3 and SKOV3GL-G4 cells compared to other SKOV3 cells (Fig. 4a, bottom). Furthermore, the epithelial cell marker E-cadherin was sequentially reduced from SKOV3GL to SKOV3GL-G4 cells, while N-cadherin, a marker of epithelial-to-mesenchymal transition, was increased (Suppl. Fig. 5a). As phospho-PHB at Y259 and T258 in the cell membrane correlated with the invasiveness of cervical and lung cancer [29], we then examined the expression of phospho-PHB in SKOV3GL-G1 to -G4 cells. The levels of phospho-PHB^Y259^, phospho-PHB^T258^, and PHB in the lipid raft domain of the plasma membrane gradually increased from SKOV3GL to SKOV3GL-G4 cells (Fig. 4b, right panel), whereas inverse correlations were found in the non-raft domain (Fig. 4b, left panel).

**Fig. 5:**
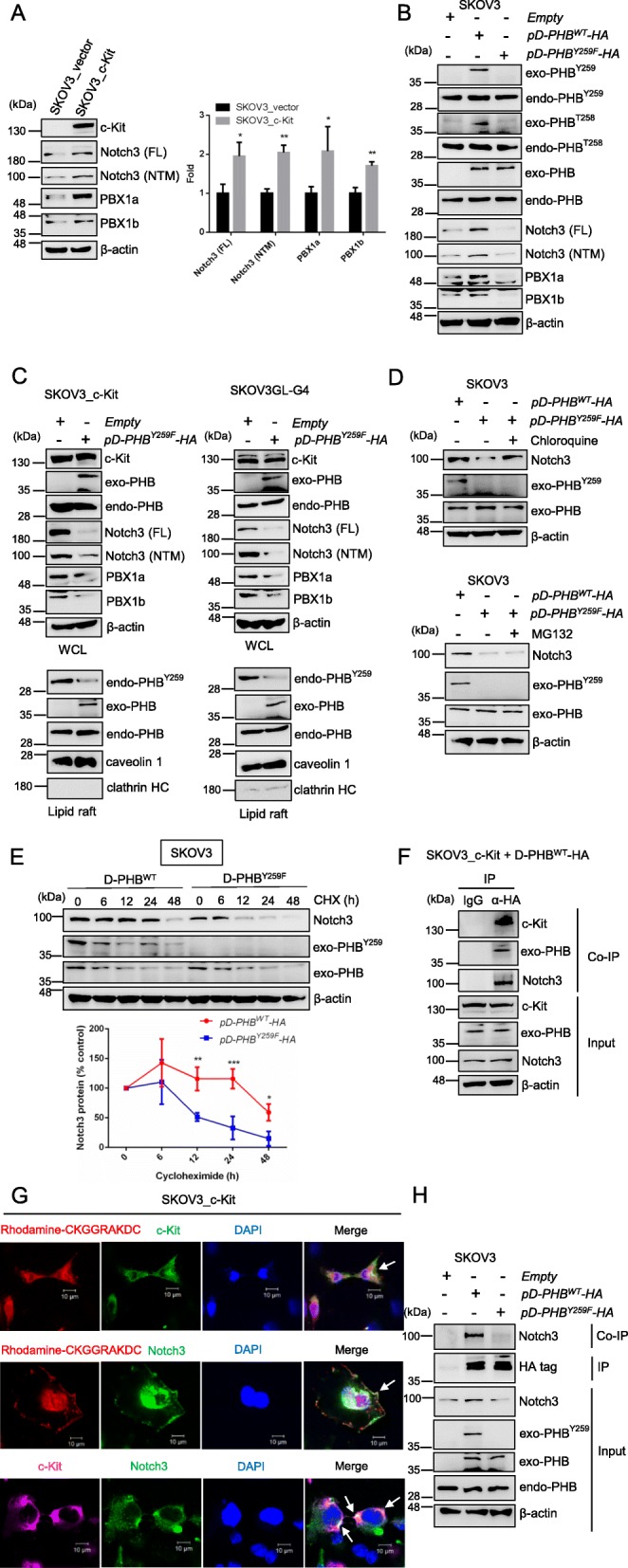
c-Kit-mediated phospho-PHB^Y259^ in the membrane raft associates with Notch3 to regulate the Notch3—PBX1 signaling pathway. **a** Analysis of Notch3 (FL & NTM) and PBX1 protein expression in SKOV3_vector and SKOV3_c-Kit cells by western blotting. The quantification data represent the mean ± SD of three independent experiments. **, *P* < 0.01; *, *P* < 0.05, *t* test. **b** SKOV3 cells were transfected with empty vector, wild-type *pD-PHB-HA*, or mutant PHB plasmid (*pD-PHB*^*Y259F*^*-HA*) for 48 h. The levels of Notch3 (FL & NTM) and PBX1 were analyzed by western blotting. **c** Transfection with empty vector or mutant PHB plasmid (*pD-PHB*^*Y259F*^*-HA*) in SKOV3_c-Kit or SKOV3GL-G4 cells for 48 h. The levels of Notch3 (FL & NTM) and PBX1 were analyzed in whole cell lysate (WCL, top). Endo-PHB^Y259^, PHB and exo-PHB expression was analyzed in the lipid raft domain (bottom) by western blotting. **d** SKOV3 cells transfected with mutant PHB plasmid (*pD-PHB*^*Y259F*^*-HA*) were treated with or without chloroquine (25 μM) (or MG132; 20 μM), and the level of Notch3 was determined by western blotting and compared to that in SKOV3 cells transfected with wild-type *pD-PHB-HA* plasmid. **e** To monitor protein stability, SKOV3 cells transfected with wild-type *pD-PHB-HA* or mutant PHB plasmid (*pD-PHB*^*Y259F*^*-HA*) were treated with cycloheximide (CHX; 50 μM), and the level of Notch3 was measured at the indicated time points. Data represent the mean ± SD of three independent experiments. ***, *P* < 0.001; **, *P* < 0.01; *, *P* < 0.05, *t* test. **f** SKOV3_c-Kit cells were transiently transfected with plasmid *pD-PHB-HA,* and immunoprecipitation was performed with anti-HA tag antibody. Coimmunoprecipitation (Co-IP) proteins were detected by western blotting with anti-c-Kit, anti-PHB, and anti-Notch3 antibodies. **g** Localization of c-Kit, raft-PHB, and Notch3 was analyzed in SKOV3_c-Kit cells through confocal microscopy. Scale bar = 10 μm. **h** SKOV3 cells were transfected with empty vector, wild-type *pD-PHB-HA*, or mutant PHB plasmid (*pD-PHB*^*Y259F*^*-HA*) for 48 h. PHB was immunoprecipitated with anti-HA antibody, and coimmunoprecipitation of Notch3 was detected by western blotting. Blots are representative of three independent experiments

Since c-Kit and Notch3 have been reported to play a role in the regulation of ovarian CSCs [19, 34, 35] and CSC-driven metastasis [36, 37], we examined their expression in these different phenotypes of SKOV3GL cells. Our results showed that c-Kit, full-length (FL) Notch3 and membrane-tethered Notch3 fragment (NTM) expression were all significantly increased in SKOV3GL-G3 and SKOV3GL-G4 cells (Fig. 4c). Moreover, knockdown of c-Kit in SKOV3GL-G4 cells reduced phospho-PHB^Y259^, phospho-PHB^T258^, PHB, and Notch3 (FL & NTM) protein expression (Fig. 4d).

Further examination showed that SKOV3GL-G3 and -G4 cells had higher clonogenic efficiency than SKOV3GL parental cells (Fig. 4e). In addition, culture of SKOV3GL and SKOV3GL-G4 cells with CSC medium on ultralow attachment plates in sphere forming assays revealed that SKOV3GL-G4 cells tended to form oncospheres more easily than SKOV3GL cells, indicating that the highly metastatic ovarian cancer cells have increased CSCs (Suppl. Fig. 5b). Moreover, SKOV3GL-G4 spheroids expressed higher levels of the stem cell markers Oct4, Nanog and SOX2 than did SKOV3GL parental cells (Fig. 4f).

We further performed an in vivo tumor initiating assay to detect tumorigenic ability. Respective subcutaneous injections of the same amount of sphere cells derived from SKOV3GL and SKOV3GL-G4 into the right flank and left flank of female nude mice showed that SKOV3GL-G4 cells formed tumors more easily than SKOV3GL cells, as indicated by bioluminescence imaging quantification (Fig. 4g). Taken together, these data demonstrated that upregulation of c-Kit and phospho-PHB in the lipid raft of more migratory phenotype(s) of SKOV3GL cells is associated with an increase in Notch3 as well as an increase in cell stemness and tumorigenesis.

### Phospho-PHB^Y259^ in the membrane raft is required for the c-Kit-mediated activation of Notch3, PBX1, β-catenin and ABCG2

Activation of Notch signaling pathways might upregulate the downstream targets of Notch, such as PBX1, to drive the stemness and carcinogenesis of cancer [38, 39]. We thus examined the effects of c-Kit on Notch3 and PBX1 in SKOV3 cells. Our results showed that overexpressing c-Kit increased the levels of Notch3 (FL & NTM), PBX1a and PBX1b (Fig. 5a). Since c-Kit interacts with PHB to induce phospho-PHB^Y259^ (Fig. 3), we then examined the role of phospho-PHB^Y259^ in mediating the stemness-related pathway Notch3—PBX1. Transfection of SKOV3 cells with the *pD-PHB*^*WT*^*-HA* plasmid that expressed exogenous PHB and phospho-PHB in the membrane raft increased Notch3 (FL & NTM) and PBX1 protein expression, whereas transfection with the mutant *pD-PHB*^*Y259F*^*-HA* plasmid did not (Fig. 5b & Suppl. Fig. 6a). On the other hand, we also confirmed that downregulation of raft-phospho-PHB^Y259^ reduced raft-phospho-PHB^T258^ expression (Fig. 5b), as we reported previously [29]. Interestingly, transfection with *pD-PHB*^*Y259F*^*-HA* in SKOV3_c-Kit or SKOV3GL-G4 cells suppressed stemness-related protein expression regardless of c-Kit overexpression in these cells (Fig. 5c & Suppl. Fig. 6b). We next examined whether the reduction of Notch3 was due to enhanced proteasomal or lysosomal degradation by using MG132 (a proteasome inhibitor) and chloroquine (a lysosome inhibitor). Our results showed that chloroquine, but not MG132, treatment rescued the Notch3 protein level in raft-phospho-PHB^Y259^-depleted cells (Fig. 5d), suggesting that downregulation of raft-phospho-PHB^Y259^ increased Notch3 degradation through the lysosomal pathway. To further confirm whether depletion of raft-phospho-PHB^Y259^ could affect the stability of Notch3, we conducted a protein degradation experiment in cells treated with cycloheximide (CHX). Notch3 protein was stable over a course of 24 h in SKOV3 cells transfected with *pD-PHB*^*WT*^*-HA,* whereas the stability of Notch3 was significantly decreased in raft-phospho-PHB^Y259^-depleted cells (Fig. 5e). Collectively, these results demonstrate that c-Kit increases raft-phospho-PHB^Y259^ to stabilize Notch3 protein, resulting in activation of the Notch3—PBX1 signaling pathway.

To examine whether c-Kit may phosphorylate PHB at Y259 to recruit Notch3 in the membrane raft to facilitate Notch3-mediated signaling in ovarian cancer, we performed a coimmunoprecipitation assay using anti-HA antibodies and found that not only c-Kit but also Notch3 were coimmunoprecipitated with raft-PHB (Fig. 5f). Confocal microscopy analysis also showed that these three components (c-Kit, raft-PHB and Notch3) were colocalized at the cell surface (Fig. 5g). Moreover, cells expressing wild-type raft-PHB protein or Y259F mutant raft-PHB protein were subjected to a coimmunoprecipitation assay. Our results showed that wild-type raft-PHB protein coimmunoprecipitated with Notch3, whereas the Y259F mutant raft-PHB protein did not (Fig. 5h). Collectively, these results demonstrate that the c-Kit-mediated increase in phospho-PHB^Y259^ stabilized Notch3 in the membrane raft, leading to activation of Notch3 signaling and upregulation of its stemness transcriptional target, PBX1.

**Fig. 6:**
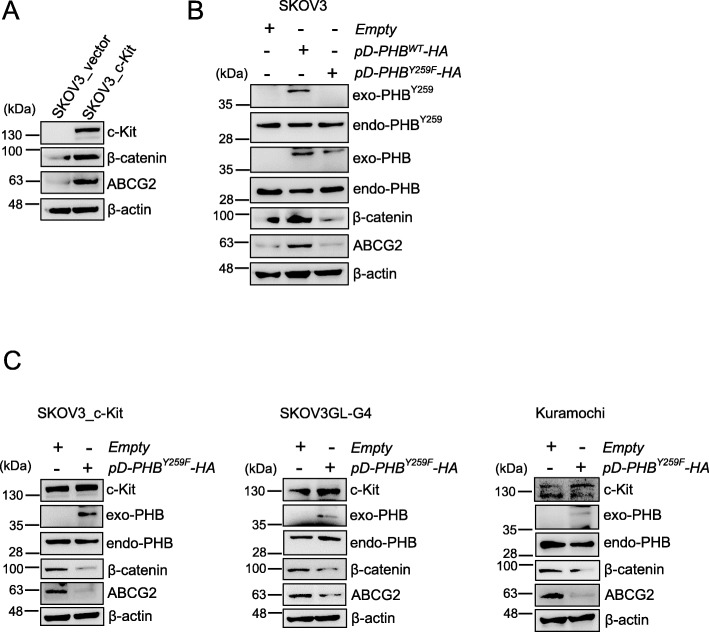
Phosphorylated PHB at Y259 by c-Kit in the membrane raft upregulates β-catenin—ABCG2 signaling. **a** Analysis of β-catenin and ABCG2 protein expression in SKOV3 and SKOV3_c-Kit cells by western blotting. **b** SKOV3 cells were transfected with empty vector, wild-type *pD-PHB-HA*, or mutant PHB plasmid (*pD-PHB*^*Y259F*^*-HA*) for 48 h. The levels of β-catenin and ABCG2 were analyzed by western blotting. **c** Transfection with empty vector or mutant PHB plasmid (*pD-PHB*^*Y259F*^*-HA*) in SKOV3_c-Kit, SKOV3GL-G4 or KURAMOCHI cells for 48 h. The levels of β-catenin and ABCG2 were analyzed by western blotting. Blots are representative of three independent experiments

Activation of c-Kit has also been reported to increase WNT/β-catenin signaling and the expression of downstream targets of β-catenin, such as ABCG2, to regulate ovarian tumor-initiating capacity and chemoresistance [19]. Our results showed that the levels of β-catenin and ABCG2 in SKOV3_c-Kit cells were higher than those in control cells (SKOV3_vector) (Fig. 6a). To examine whether this effect of c-Kit was dependent on phospho-PHB, SKOV3 cells were transfected with *pD-PHB*^*WT*^*-HA* and *pD-PHB*^*Y259F*^*-HA* plasmids. Overexpression of phospho-PHB in the membrane raft of SKOV3 cells by *pD-PHB*^*WT*^*-HA* enhanced β-catenin and ABCG2 protein expression, whereas transfection with *pD-PHB*^*Y259F*^*-HA* did not (Fig. 6b & Suppl. Fig. 6c). In comparison, suppression of phospho-PHB^Y259^ by transfection with *pD-PHB*^*Y259F*^*-HA* in c-Kit-overexpressing tumor cells, such as SKOV3_c-Kit, SKOV3GL-G4 or KURAMOCHI cells, reduced the levels of β-catenin and ABCG2 (Fig. 6c & Suppl. Fig. 6d). Taken together, these results suggest that the c-Kit-mediated increase in phospho-PHB^Y259^ activates Notch3 and β-catenin and upregulates their transcriptional targets, PBX1 and ABCG2, respectively.

### Phospho-PHB^Y259^ in the membrane raft is required for ovarian CSC phenotypes, tumorigenicity and drug resistance

**Fig. 7:**
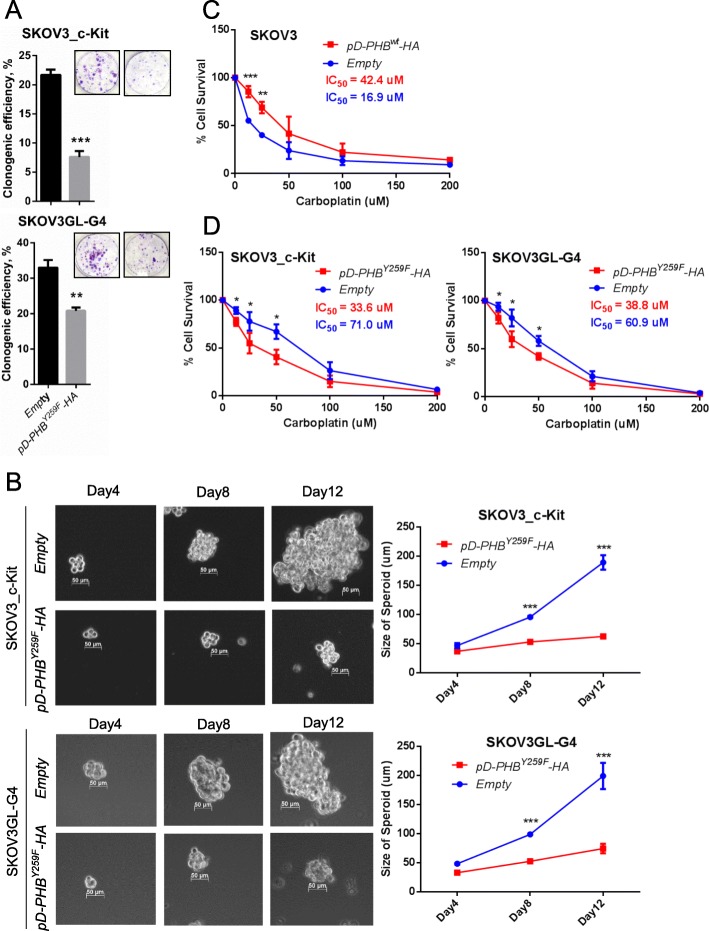
Phospho-PHB^Y259^ in the membrane raft promotes ovarian cancer stem cell survival and potentiates drug-resistant phenotypes. **a** SKOV3_c-Kit and SKOV3GL-G4 cells were transfected with empty vector or mutant PHB plasmid (*pD-PHB*^*Y259F*^*-HA*) for 48 h and plated at a density of 300 cells/well in 6-well plates for 14 days. The colony formation was visualized with crystal violet. **b** Spheroid-forming assay in SKOV3_c-Kit (or SKOV3GL-G4) cells transfected with empty vector or *pD-PHB*^*Y259F*^*-HA* plasmid. Representative photos were taken on days 4, 8, and 12. Spheroid sizes were digitally determined. **c** Carboplatin sensitivity assay. SKOV3 cells were transfected with empty vector or wild-type *pD-PHB-HA* for 48 h. Cells were treated with carboplatin for 72 h, and cell viability was detected. **d** SKOV3_c-Kit and SKOV3GL-G4 cells were transfected with empty vector or *pD-PHB*^*Y259F*^*-HA* plasmid. After treatment with carboplatin for 72 h, cell viability was measured using the CCK-8 Assay Kit. The above data represent the mean ± SD of three independent experiments. ***, *P* < 0.001; **, *P* < 0.01; *, *P* < 0.05, *t* test

To determine the effects of phospho-PHB^Y259^ on CSC phenotypes and tumorigenicity, SKOV3_c-Kit and SKOV3GL-G4 cells were transfected with *pD-PHB*^*Y259F*^*-HA* and empty vector control. Downregulation of phospho-PHB^Y259^ by *pD-PHB*^*Y259F*^*-HA* transfection reduced the stemness of SKOV3_c-Kit or SKOV3GL-G4 cells compared to that of control cells, as indicated by a decrease in their colony and spheroid forming abilities (Fig. 7a, b). Since chemoresistance is a hallmark of CSCs, we then evaluated whether changes in phospho-PHB^Y259^ expression in the membrane raft can affect the responses of ovarian cancer cells to carboplatin, a chemotherapeutic drug commonly used in treating ovarian cancer patients. Cell viability assays showed that ectopic wild-type PHB expression in the membrane raft domain increased the resistance of SKOV3 cells to carboplatin (IC_50_ 42.4 vs 16.9 μM; Fig. 7c). On the other hand, downregulating phospho-PHB^Y259^ by transfection of SKOV3_c-Kit and SKOV3GL-G4 cells with mutant *pD-PHB*^*Y259F*^*-HA* reduced their resistance to carboplatin, as indicated by a decrease in IC_50_ (Fig. 7d).

**Fig. 8:**
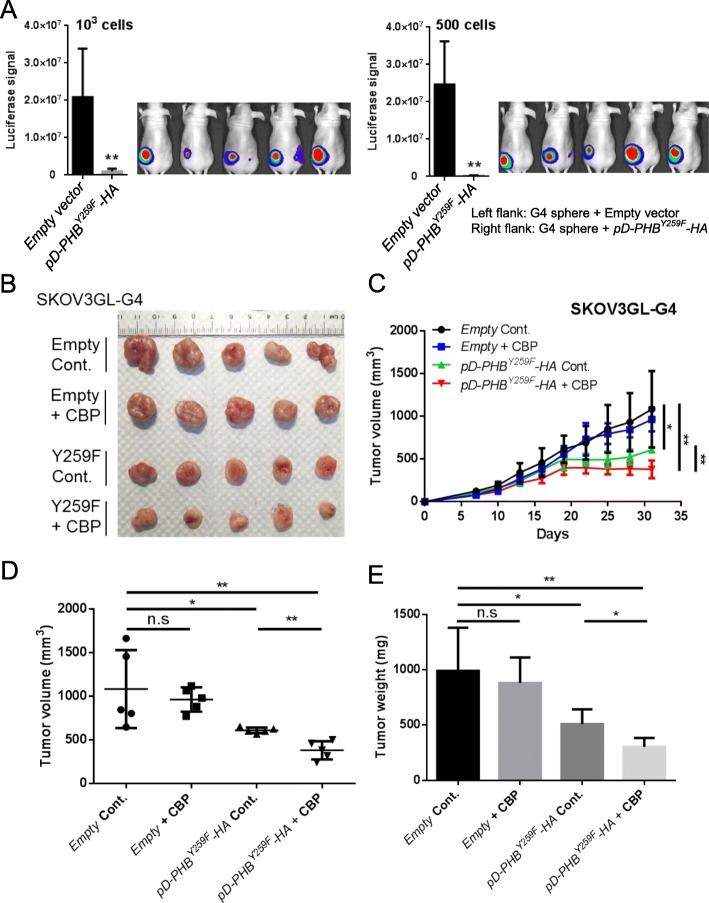
Downregulation of raft-phospho-PHB^Y259^ reduces tumorigenic ability and sensitizes tumors to carboplatin treatment. **a** SKOV3GL-G4 cells were transfected with empty vector or mutant PHB plasmid (*pD-PHB*^*Y259F*^*-HA*) for 48 h. After transfection, cells were cultured in CSC medium for 12 days. After 12 days, 1000 or 500 cells in 50 μl DPBS were mixed with 50 μl Matrigel and subcutaneously injected into the flanks of nude mice. In vivo CSC properties were determined by luciferase signal at week 4 post injection (mean ± SD; *n* = 5 per group; **, *P* < 0.01; *, *P* < 0.05, *t* test). **b** SKOV3GL-G4 cells were transfected with empty vector or *pD-PHB*^*Y259F*^*-HA* for 48 h. After transfection, 2 × 10^6^ cells in 50 μl DPBS were mixed with 50 μl Matrigel and subcutaneously injected into flanks of nude mice. Treatment with carboplatin (CBP) began 1 week after injection. Representative tumor images excised from mice at the end of the experiment at day 31. **c** Tumor growth kinetics were measured every 3 days after carboplatin (CBP) treatment. **d, e** Endpoints of tumor volume and tumor weight were measured (mean ± SD; n = 5 per group; **, *P* < 0.01; *, *P* < 0.05, *t* test)

To further examine whether raft-phospho-PHB^Y259^ is required for CSC tumor formation in vivo, SKOV3GL-G4 sphere cells were transfected with *pD-PHB*^*Y259F*^*-HA* or empty vector and then subcutaneously injected into the right or left flank of nude mice. The results showed that suppression of phospho-PHB^Y259^ by *pD-PHB*^*Y259F*^*-HA* transfection decreased tumor formation in vivo (Fig. 8a). We then examined the effect of raft-phospho-PHB^Y259^ downregulation in combination with carboplatin treatment in vivo. A representative image of the excised tumors on day 31 after inoculation is presented in Fig. 8b. As shown in Fig. 8c, treatment with carboplatin in SKOV3GL-G4 cells did not markedly reduce the tumor growth rate, whereas suppression of raft-phospho-PHB^Y259^ in SKOV3GL-G4 cells significantly reduced the tumor growth rate. Combination treatment of SKOV3GL-G4 cells with carboplatin and downregulation of raft-phospho-PHB^Y259^ showed additive cytotoxic effects. After 31 days, the tumor volume and tumor weight formed in the *pD-PHB*^*Y259F*^*-HA* combination with carboplatin group were significantly reduced compared with those in the empty vector control group and *pD-PHB*^*Y259F*^*-HA* group. (Fig. 8d, e). Taken together, these results indicate that the c-Kit-mediated increase in phospho-PHB^Y259^ plays an important role in the stemness, tumorigenicity and drug resistance of ovarian cancer in vitro and in vivo.

## Discussion

The eradication of ovarian CSCs is generally considered to be an important prerequisite for preventing cancer metastasis and relapse [40]. Although abnormal overexpression of phospho-PHB and c-Kit, a type III receptor tyrosine kinase (RTK), is known to increase metastasis of cancer [22, 29], the interrelationship between phospho-PHB and c-Kit in CSCs and cancer metastasis is unclear. Here, we found that the association of activated c-Kit with PHB-induced phospho-PHB^Y259^ in the membrane raft domain (Figs. 1, 2, 3) plays an important role in ovarian cancer stemness and drug resistance. Of note, since c-Kit is a tyrosine kinase, it can phosphorylate not only tyrosine 259 on PHB but also other tyrosine residues, such as tyrosine 114 and 249. Stimuli, notably epidermal growth factor, insulin (and insulin-like growth factor) and platelet-derived growth factor, have previously been shown to bind type I RTKs, type II RTKs and type III RTKs, respectively, to induce phosphorylation of PHB at one or more tyrosine residues 114, 249, 259 [41–44]. Our findings thus identified c-Kit as another RTK that induces phospho-PHB and may help shed light on the effects of RTKs and facilitate the elucidation of the mechanism of action of RTKs on PHB. It would be interesting, for instance, to elucidate whether and how phosphorylation of PHB by c-Kit is similar to or distinguishable from other types of RTK, such as epidermal growth factor receptor and insulin receptor.

Although c-Kit has been reported to collaborate with Notch signaling in regulating multiple cellular functions [45–47], it is unclear whether c-Kit can regulate or activate Notch3, which plays an important role in CSC [34, 35, 48, 49] and high-grade ovarian serous carcinoma [50–53]. In our study, we found that c-Kit, Notch3 and raft-phospho-PHB protein expression were enhanced in our generated metastatic phenotypes of SKOV3 cells (Fig. 4a-c). Knockdown of c-Kit reduced the levels of phospho-PHB and Notch3 in metastatic ovarian cancer cells (Fig. 4d). These findings imply that c-Kit may activate Notch3 through the induction of phospho-PHB in the membrane raft domain. In Fig. 5, we clearly demonstrated that c-Kit upregulates phospho-PHB^Y259^ in the lipid raft domain to interact with the membrane-tethered Notch3 fragment and enhance the Notch3—PBX1 signaling pathway in ovarian cancer cells. However, whether this increase in PBX1, a stem cell reprogramming factor in ovarian cancer chemoresistance [39], is primarily responsible for the drug resistance of ovarian cancer cells to carboplatin (shown in Fig. 7) or requires the cooperation of other factor(s) remains to be elucidated.

In this study, we found that PHB was associated with c-Kit and Notch3 in the lipid raft domain. Furthermore, downregulation of raft-phospho-PHB^Y259^ decreased the association between c-Kit-mediated phospho-PHB^Y259^ and Notch3 and increased Notch3 degradation through the lysosomal pathway, leading to a reduction in PBX1 expression (Fig. 5). However, whether raft-phospho-PHB^Y259^ directly regulates Notch3 or requires the involvement of other mediators (or co-factors) still needs to be further investigated.

It has been reported that c-Kit regulates the chemoresistance and tumor-initiating capacity of ovarian cancer cells through the β-catenin—ABCG2 pathway [19]. In our study, we found that raft-phospho-PHB^Y259^ is similar to a bridge that connects c-Kit and β-catenin—ABCG2 signaling. Raft-phospho-PHB^Y259^ is required for the β-catenin-mediated upregulation of ABCG2 (Fig. 6). In our study, the c-Kit-mediated upregulation of cancer stemness-related Notch3—PBX1 and β-catenin—ABCG2 signaling pathways and promotion of ovarian cancer tumorigenicity and drug resistance were all phospho-PHB^Y259^ dependent. A proposed model of c-Kit-induced PHB phosphorylation and activation of the Notch3 and β-catenin signaling pathways is shown in Suppl. Fig. 7. These findings provide new insight into raft-phospho-PHB^Y259^-mediated regulation of stemness and chemoresistance phenotypes in ovarian cancer and perhaps other cancers.

Despite many recent advances in ovarian cancer therapies, ultimately, metastatic ovarian cancer still recurs and progresses to incurable lethal disease. Here, we found that phospho-PHB^Y259^ in the membrane raft domain acted as an adaptor molecule to initiate and transmit stemness and drug resistance signals in ovarian cancer cells. Inhibition of raft-phospho-PHB^Y259^ reduced tumorigenicity and resensitized cancer cells to carboplatin treatment. These results indicate that the c-Kit/raft-phospho-PHB^Y259^ axis could play a pivotal role in regulating the chemosensitivity of ovarian cancer towards carboplatin. Further studies focusing on targeting c-Kit/raft-phospho-PHB^Y259^ are warranted.

## Conclusions

In summary, we have shown that the association of c-Kit with PHB upregulated phospho-PHB^Y259^ in the lipid raft domain, resulting in subsequent activation of the Notch3—PBX1 and β-catenin—ABCG2 signaling pathways to increase stemness, tumorigenicity and drug resistance in ovarian cancer. Interventions targeting c-Kit and phospho-PHB^Y259^ might be used for screening and development of new targeting therapeutics to treat advanced ovarian cancer and other related diseases.

## Supplementary information

**Additional file 1 : Figure S1.** Group-based Prediction System (GPS) online software predicted that three tyrosine kinases, c-Kit, PDGFR, and EphA3, may phosphorylate PHB at tyrosine 259. **Figure S2.** The phosphorylated PHB protein from the kinase reaction was proteolytically digested and analyzed using liquid chromatography-tandem mass spectrometry (LC/MS/MS). **Figure S3.** Kaplan-Meier curves of overall survival of *c-Kit* and *PHB* expression from the KM-Plotter database. **Figure S4.** Evaluation of the level of PHB or c-Kit in the plasma membrane by confocal fluorescence microscopy. **Figure S5.** Advanced orthotopic ovarian cancer cells enhanced epithelial-to-mesenchymal transition and exhibited CSC phenotypes. **Figure S6.** Overexposed blots of phospho-PHB and PHB in SKOV3, SKOV3_c-Kit, SKOV3GL-G4 and Kuramochi cells. **Figure S7.** A proposed model to illustrate the mechanism by which c-Kit-mediated phospho-PHB^Y259^ results in subsequent activation of the Notch3 and β-catenin signaling pathways. **Table S1.** Patient specifications of the human ovarian cancer tissue array. **Table S2.** Primer sequences used to amplify specific target genes.

## Data Availability

All data generated or analyzed during this study are included in this published article.

## Abbreviations

CBP: Carboplatin
CHX: Cycloheximide
c-Kit: mast/stem cell factor receptor
CSCs: Cancer stem cells
DPBS: Dulbecco’s phosphate-buffered saline
DTT: Dithiothreitol
EMT: Epithelial mesenchymal transition
FL: Full-length
GL: Green fluorescent protein and luciferase
GPS: Group-based Prediction System
HA: Hemagglutinin
HRP: Horseradish peroxidase
LC/MS/MS: Liquid chromatography-tandem mass spectrometry
NTM: Membrane-tethered Notch3 fragment
PHB: Prohibitin
RTK: Receptor tyrosine kinase
SCF: Stem cell factor
siRNA: Small interfering RNA
TBST: Tris-buffered saline with Tween 20
TMA: Tissue microarray
WT: Wild type
