# Supplementary material for: A novel c-Kit/phospho-prohibitin axis enhances ovarian cancer stemness and chemoresistance via Notch3—PBX1 and β-catenin—ABCG2 signaling
Source: J Biomed Sci. 2020 Mar 13;27:42. doi: 10.1186/s12929-020-00638-x (PMC7071647; doi:10.1186/s12929-020-00638-x)
Supplement: Supplementary file 1 — Additional file 1 : Figure S1. Group-based Prediction System (GPS) online software predicted that three tyrosine kinases, c-Kit, PDGFR, and EphA3, may phosphorylate PHB at tyrosine 259. Figure S2. The phosphorylated PHB protein from the kinase reaction was proteolytically digested and analyzed using liquid chromatography-tandem mass spectrometry (LC/MS/MS). Figure S3. Kaplan-Meier curves of overall survival of c-Kit and PHB expression from the KM-Plotter database. Figure S4. Evaluation of the level of PHB or c-Kit in the plasma membrane by confocal fluorescence microscopy. Figure S5. Advanced orthotopic ovarian cancer cells enhanced epithelial-to-mesenchymal transition and exhibited CSC phenotypes. Figure S6. Overexposed blots of phospho-PHB and PHB in SKOV3, SKOV3_c-Kit, SKOV3GL-G4 and Kuramochi cells. Figure S7. A proposed model to illustrate the mechanism by which c-Kit-mediated phospho-PHBY259 results in subsequent activation of the Notch3 and β-catenin signaling pathways. Table S1. Patient specifications of the human ovarian cancer tissue array. Table S2. Primer sequences used to amplify specific target genes. [file 12929_2020_638_MOESM1_ESM.docx]

Supplementary Information

**A novel c-Kit/phospho-prohibitin axis enhances ovarian cancer stemness and chemoresistance via Notch3—PBX1 and β-catenin—ABCG2 signaling**

Chia-Hsun Fang^1,3^, Yi-Te Lin^1^, Chi-Ming Liang^2^, Shu-Mei Liang^1,3^

^1^Agricultural Biotechnology Research Center, ^2^Genomics Research Center, Academia Sinica, 128 Academia Rd, Sec. 2, Taipei 11529, Taiwan

^3^Institute of Biotechnology, National Taiwan University, 4F, No. 81, Chang-Xing St., Taipei 10672, Taiwan

**Corresponding author**: Dr. S-M Liang, Agricultural Biotechnology Research Center, Academia Sinica, 128 Academia Rd, Sec. 2, Taipei 11529, Taiwan, Tel: 886-2-2787-2082; E-mail: [smyang@gate.sinica.edu.tw](mailto:smyang@gate.sinica.edu.tw)

**This file includes:**

Figs. S1 to S7

Tables S1 to S2


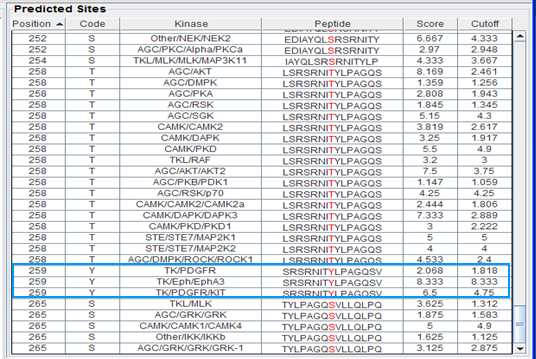


**Fig. S1.** Group-based Prediction System (GPS) online software predicted that three tyrosine kinases, c-Kit, PDGFR, and EphA3, may phosphorylate PHB at tyrosine 259. According to their score and cutoff, c-Kit (KIT) is the most likely protein to phosphorylate PHB at Y259.


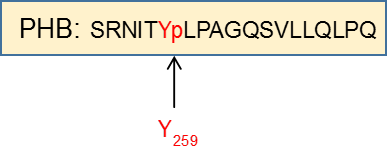


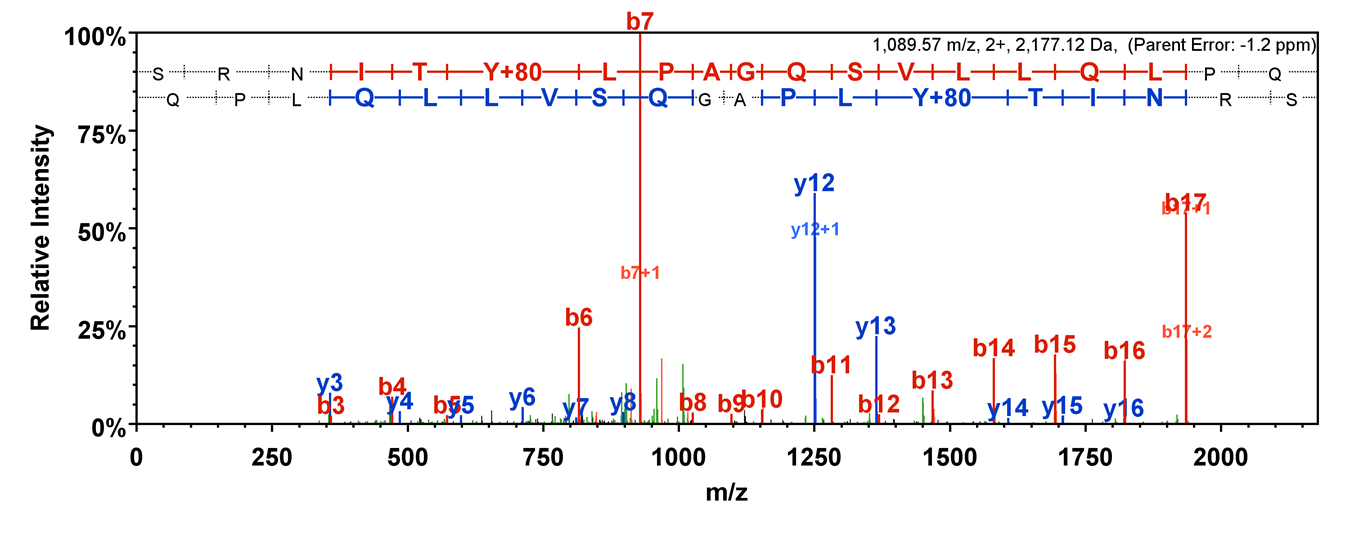


**Fig. S2.** The phosphorylated PHB protein from the kinase reaction was proteolytically digested and analyzed using liquid chromatography-tandem mass spectrometry (LC/MS/MS). The MS/MS spectrum showed PHB was phosphorylated at tyrosine 259.

**A**


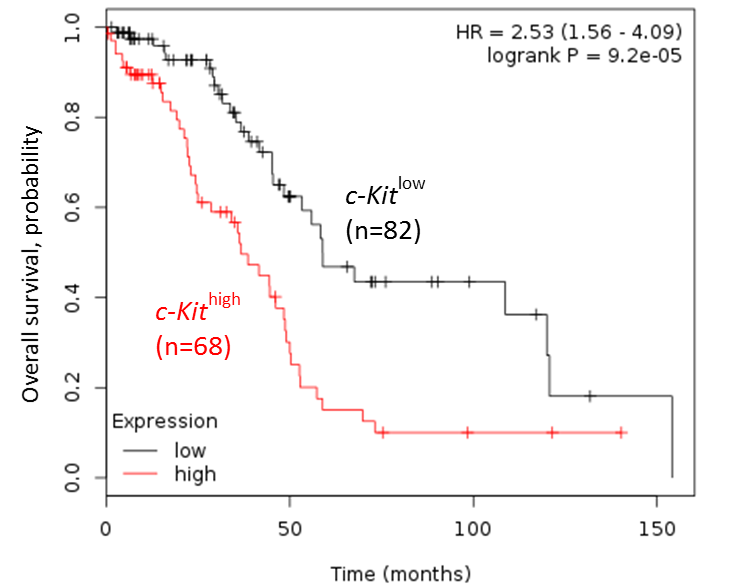


**B**


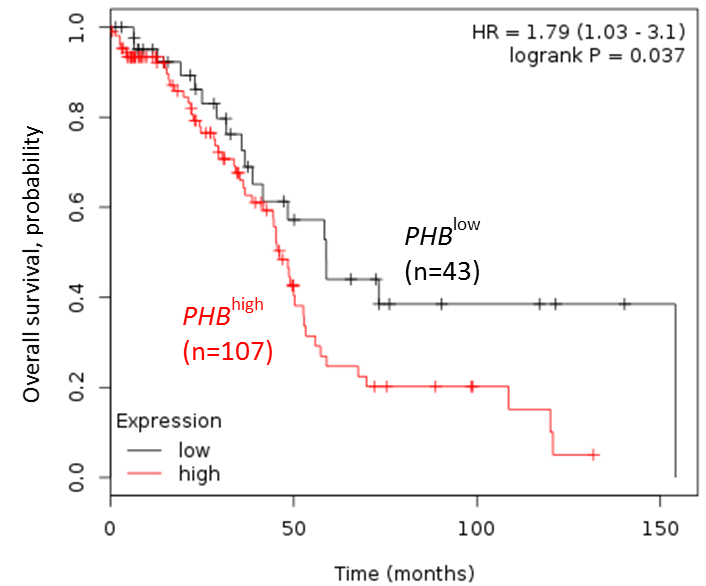


**Fig. S3.** Kaplan-Meier curves of overall survival of *c-Kit* and *PHB* expression in a cohort of optimally debulked ovarian serous carcinoma patients from the KM-Plotter database.


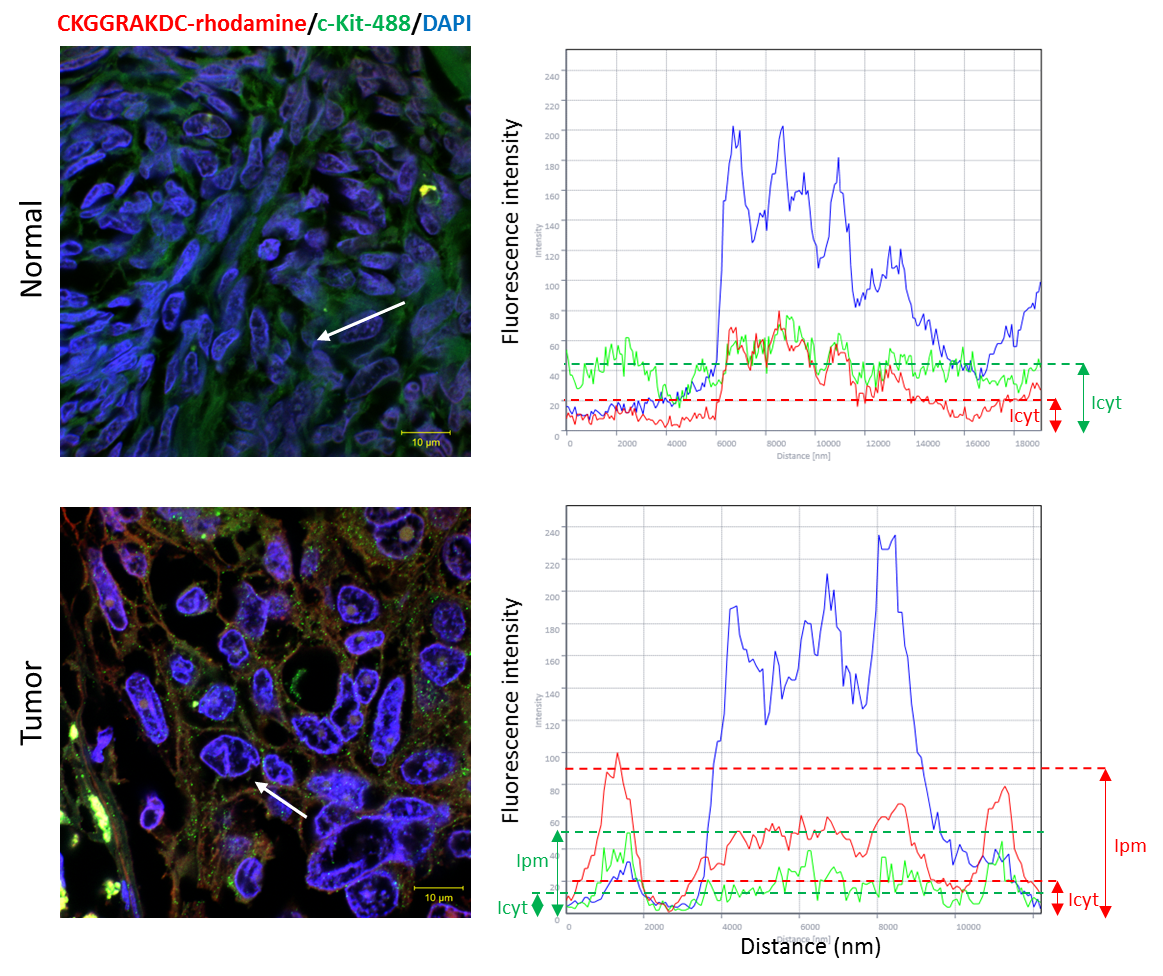


**Fig. S4.** Evaluation of the level of PHB or c-Kit in the plasma membrane by confocal fluorescence microscopy. Human ovarian normal and cancer tissue sections were stained using rhodamine-tagged CKGGRAKDC (red) for PHB, Alexa488 (green) for c-Kit, DAPI (blue) for nuclei and viewed under a confocal fluorescence microscopy. The cellular distribution of PHB or c-Kit as indicated by fluorescent intensity across the region marked by the white arrow (left panel) was obtained by using ZEN analysis software and plotted as a line intensity histogram (right panel). The average ratio of fluorescence intensity of the plasma membrane (Ipm) to cytoplasm (Icyt) was used to quantitate the extent of membrane localization of PHB or c-Kit. Scale bar, 10 μm.

**A**


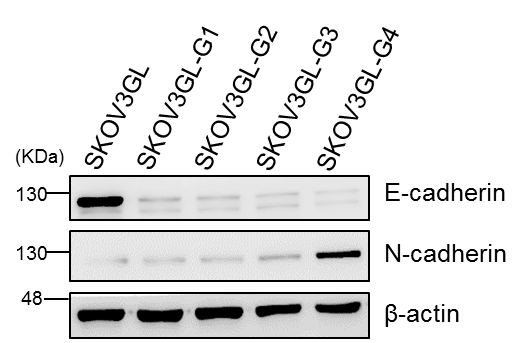


**B**

**
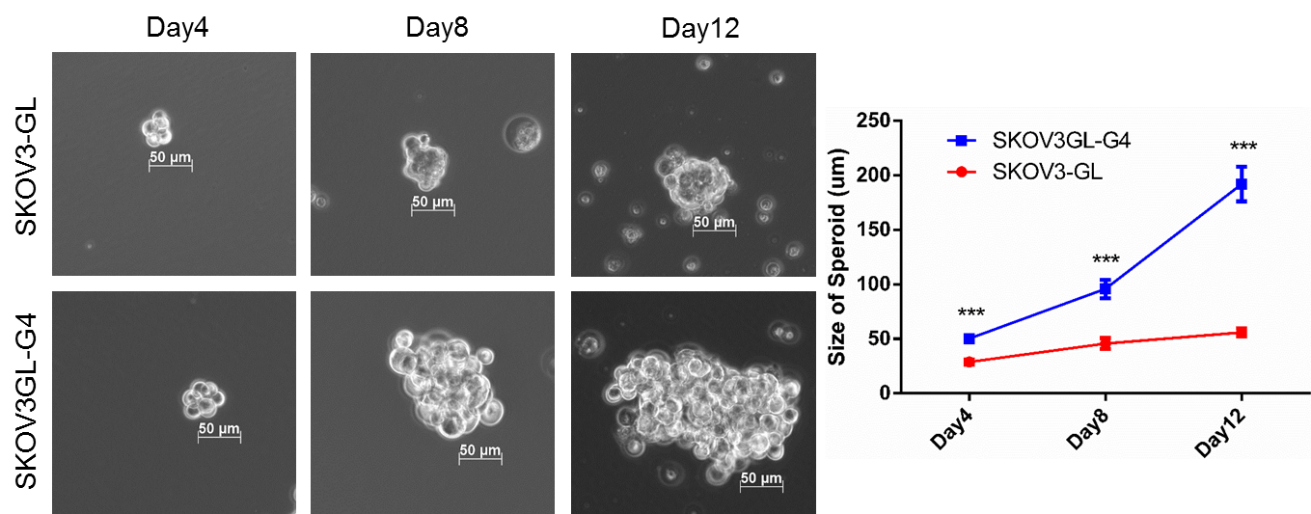
**

**Fig. S5.** Advanced orthotopic ovarian cancer cells enhanced epithelial-to-mesenchymal transition and exhibited CSC phenotypes. **(A)** Expressions of E-cadherin and N-cadherin were analyzed from SKOV3GL to SKOV3GL-G4 cells by western blot. **(B)** Spheroid-forming assay in SKOV3-GL and SKOV3GL-G4 cells. Representative photos were taken on days 4, 8, and 12. Spheroid sizes were digitally determined. ***, *P* ˂ 0.001, *t* test.

**A C**


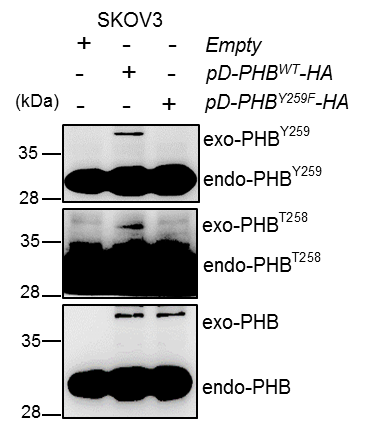

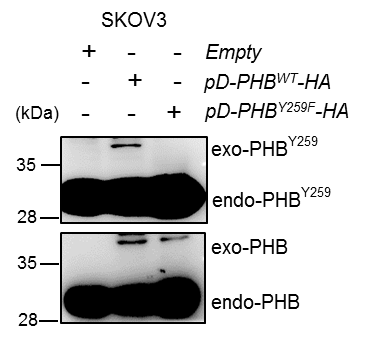


**B**


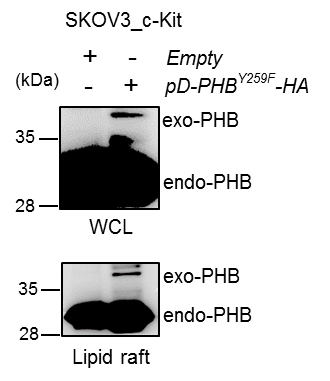

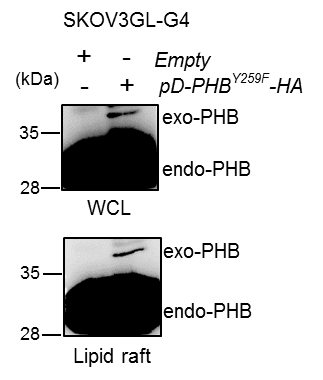


**D**


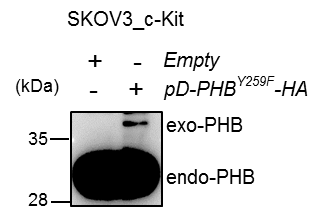

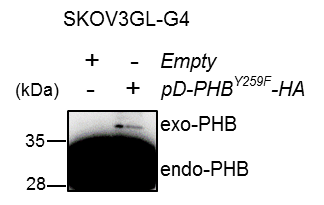


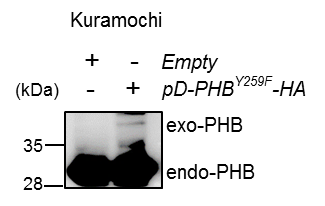


**Fig. S6.** Overexposed blots of phospho-PHB and PHB in SKOV3, SKOV3_c-Kit, SKOV3GL-G4 and Kuramochi cells.


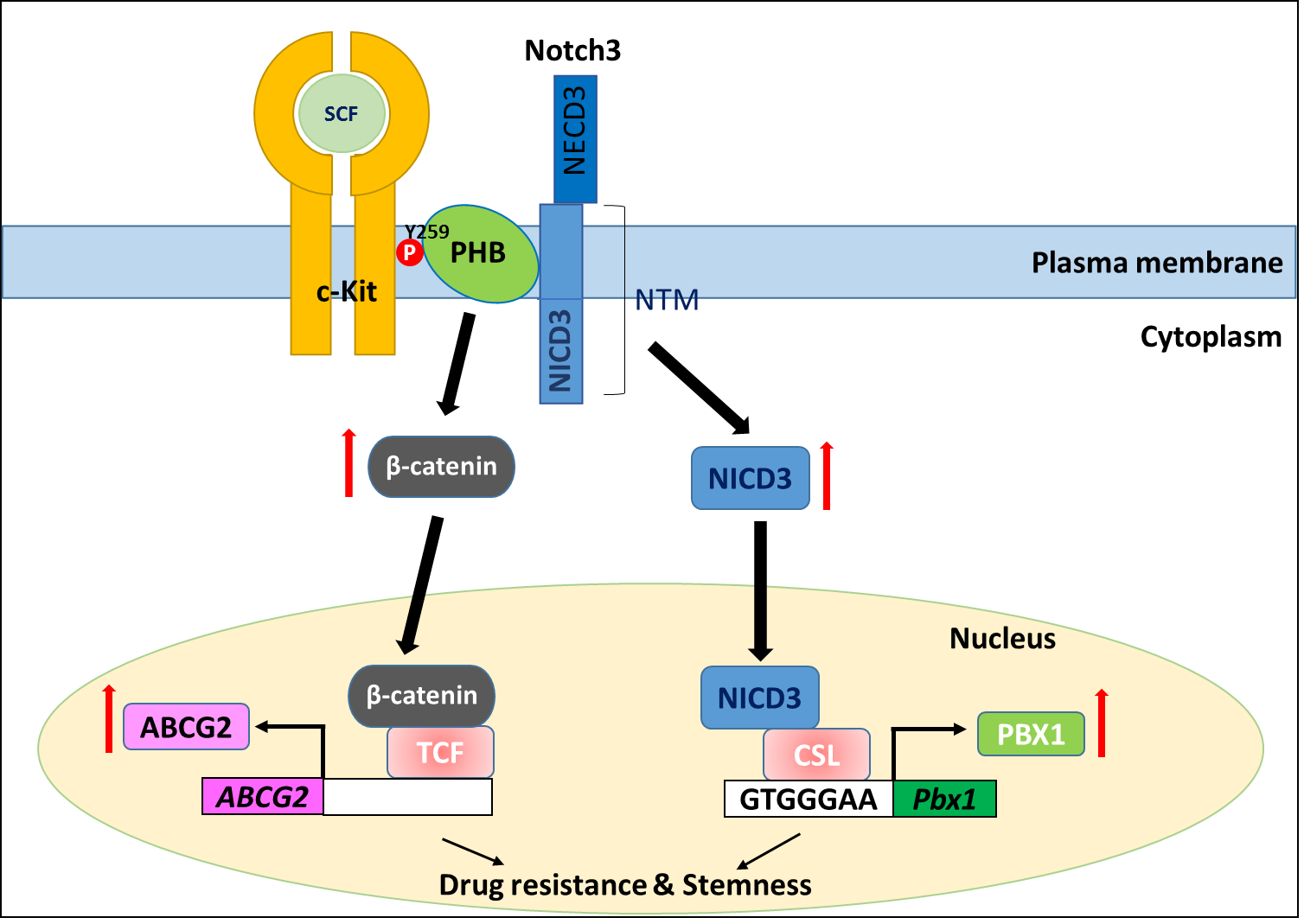


**Fig. S7.** A proposed model to illustrate the mechanism by which c-Kit-mediated phospho-PHB^Y259^ results in subsequent activation of the Notch3 and β-catenin signaling pathways.

**Table S1. Patient specifications of the human ovarian cancer tissue array.**

| **No.** | **Sex** | **Age** | **Organ** | **Pathology diagnosis** | **Stage** |
| --- | --- | --- | --- | --- | --- |
| 1 | F | 34 | Ovary | Serous papillary adenocarcinoma | Ib |
| 2 | F | 42 | Ovary | Serous papillary adenocarcinoma | I |
| 3 | F | 59 | Ovary | Serous papillary adenocarcinoma | Ia |
| 4 | F | 48 | Ovary | Serous papillary adenocarcinoma | I |
| 5 | F | 38 | Ovary | Serous papillary adenocarcinoma | IV |
| 6 | F | 47 | Ovary | Serous papillary adenocarcinoma | Ia |
| 7 | F | 25 | Ovary | Serous papillary adenocarcinoma | I |
| 8 | F | 51 | Ovary | Serous papillary adenocarcinoma | IIIc |
| 9 | F | 57 | Ovary | Serous papillary adenocarcinoma | Ic |
| 10 | F | 53 | Ovary | Serous adenocarcinoma | Ia |
| 11 | F | 61 | Ovary | Serous papillary adenocarcinoma | Ib |
| 12 | F | 60 | Ovary | Serous papillary adenocarcinoma | Ib |
| 13 | F | 32 | Ovary | Serous papillary adenocarcinoma | I |
| 14 | F | 53 | Ovary | Serous papillary adenocarcinoma | I |
| 15 | F | 33 | Ovary | Serous papillary adenocarcinoma | I |
| 16 | F | 50 | Ovary | Serous papillary adenocarcinoma | IIa |
| 17 | F | 22 | Ovary | Serous papillary adenocarcinoma | IIb |
| 18 | F | 64 | Ovary | Serous adenocarcinoma | IV |
| 19 | F | 26 | Ovary | Serous papillary adenocarcinoma | IIIc |
| 20 | F | 56 | Ovary | Serous papillary adenocarcinoma | II |
| 21 | F | 48 | Ovary | Serous adenocarcinoma | Ib |
| 22 | F | 35 | Ovary | Serous papillary adenocarcinoma | Ia |
| 23 | F | 52 | Ovary | Serous papillary adenocarcinoma | Ia |
| 24 | F | 59 | Ovary | Serous adenocarcinoma | Ic |
| 25 | F | 68 | Ovary | Serous adenocarcinoma | Ia |
| 26 | F | 46 | Ovary | Serous adenocarcinoma | Ia |
| 27 | F | 49 | Ovary | Serous adenocarcinoma | IIIc |
| 28 | F | 53 | Ovary | Serous papillary adenocarcinoma | IV |
| 29 | F | 52 | Ovary | Serous adenocarcinoma | II |
| 30 | F | 51 | Ovary | Serous adenocarcinoma | Ic |
| 31 | F | 54 | Ovary | Serous papillary adenocarcinoma | IIb |
| 32 | F | 56 | Ovary | Serous papillary adenocarcinoma | IV |
| 33 | F | 54 | Ovary | Serous papillary adenocarcinoma | IIIc |
| 34 | F | 51 | Ovary | Serous papillary adenocarcinoma | Ia |
| 35 | F | 60 | Ovary | Serous papillary adenocarcinoma | IIa |
| 36 | F | 51 | Ovary | Serous papillary adenocarcinoma | IIIc |
| 37 | F | 73 | Ovary | Serous papillary adenocarcinoma | IIa |
| 38 | F | 38 | Ovary | Serous adenocarcinoma | IIIc |
| 39 | F | 47 | Ovary | Serous papillary adenocarcinoma | Ia |
| 40 | F | 58 | Ovary | Serous papillary adenocarcinoma | I |
| 41 | F | 41 | Ovary | Serous adenocarcinoma | I |
| 42 | F | 50 | Ovary | Serous adenocarcinoma | I |
| 43 | F | 49 | Ovary | Serous papillary adenocarcinoma | II |
| 44 | F | 52 | Ovary | Serous adenocarcinoma | Ic |
| 45 | F | 46 | Ovary | Serous adenocarcinoma | IIIc |
| 46 | F | 46 | Ovary | Serous adenocarcinoma | IIIc |
| 47 | F | 57 | Ovary | Serous adenocarcinoma | IIIc |
| 48 | F | 52 | Ovary | Serous adenocarcinoma | II |
| 49 | F | 75 | Ovary | Serous adenocarcinoma | II |
| 50 | F | 54 | Ovary | Serous adenocarcinoma | Ib |
| 51 | F | 66 | Ovary | Serous adenocarcinoma | IIIc |
| 52 | F | 49 | Ovary | Serous adenocarcinoma | IIIc |
| 53 | F | 54 | Ovary | Serous adenocarcinoma | IIIc |
| 54 | F | 42 | Ovary | Serous adenocarcinoma | IIIc |
| 55 | F | 60 | Ovary | Serous papillary adenocarcinoma | Ia |
| 56 | F | 47 | Ovary | Serous adenocarcinoma | IIa |
| 57 | F | 47 | Ovary | Serous adenocarcinoma | Ic |
| 58 | F | 72 | Ovary | Serous adenocarcinoma | Ia |
| 59 | F | 55 | Ovary | Serous adenocarcinoma | II |
| 60 | F | 49 | Ovary | Serous adenocarcinoma | II |
| 61 | F | 32 | Ovary | Serous adenocarcinoma | IIIc |
| 62 | F | 60 | Ovary | Serous adenocarcinoma | Ib |
| 63 | F | 34 | Ovary | Serous papillary adenocarcinoma | Ia |
| 64 | F | 65 | Ovary | Serous papillary adenocarcinoma | IIa |
| 65 | F | 40 | Ovary | Normal ovarial tissue | - |
| 66 | F | 19 | Ovary | Normal ovarial tissue | - |
| 67 | F | 18 | Ovary | Normal ovarial tissue | - |
| 68 | F | 40 | Ovary | Normal ovarial tissue | - |
| 69 | F | 21 | Ovary | Normal ovarial tissue | - |
| 70 | F | 21 | Ovary | Normal ovarial tissue | - |
| 71 | F | 18 | Ovary | Normal ovarial tissue | - |
| 72 | F | 15 | Ovary | Normal ovarial tissue | - |
| 73 | F | 20 | Ovary | Normal ovarial tissue | - |
| 74 | F | 14 | Ovary | Normal ovarial tissue | - |

**Table S2. Primer sequences used to amplify specific target genes**

| Gene sequences | Sequences (5’→3’) | PCR product (bp) | GenBank accession no. |
| --- | --- | --- | --- |
| Oct4 | Sense CAACTCCGATGGGGCCT  Antisense CTTCAGGAGCTTGGCAAATTG | 148 | NM_002701.6 |
| Nanog | Sense CCTGTGATTTGTGGGCCTG  Antisense GACAGTCTCCGTGTGAGGCAT | 78 | NM_024865.4 |
| SOX2 | Sense TACAGCATGTCCTACTCGCAG  Antisense GAGGAAGAGGTAACCACAGGG | 110 | NM_003106.4 |
| GAPDH | Sense ATGGGGAAGGTGAAGGTCGG  Antisense GACGGTGCCATGGAATTTGC | 180 | NM_002046.7 |
